# Detection of early changes in the post-radiosurgery vestibular schwannoma microenvironment using multinuclear MRI

**DOI:** 10.1038/s41598-021-95022-6

**Published:** 2021-08-03

**Authors:** Daniel Lewis, Damien J. McHugh, Ka-loh Li, Xiaoping Zhu, Catherine Mcbain, Simon K. Lloyd, Alan Jackson, Omar N. Pathmanaban, Andrew T. King, David J. Coope

**Affiliations:** 1 Dept. of Neurosurgery, Manchester Centre for Clinical Neurosciences, Salford Royal NHS Foundation Trust, Manchester Academic Health Science Centre, Stott Lane, Salford, Greater Manchester, M6 8HD UK; 2 Geoffrey Jefferson Brain Research Centre, Manchester Academic Health Science Centre, Northern Care Alliance NHS Group, University of Manchester, Manchester, UK; 3 Division of Informatics, Imaging and Data Sciences, Wolfson Molecular Imaging Centre (WMIC), University of Manchester, Manchester, UK; 4 Division of Cancer Sciences, University of Manchester, Manchester, UK; 5 Department of Clinical Oncology, Christie NHS Foundation Trust, Manchester, UK; 6 Department of Otolaryngology, Salford Royal NHS Foundation Trust, Manchester Academic Health Science Centre, Manchester, UK; 7 Department of Otolaryngology, Manchester University NHS Foundation Trust, Manchester Academic Health Science Centre, Manchester, UK; 8 Division of Cell Matrix Biology & Regenerative Medicine, Faculty of Biology Medicine and Health, School of Biological Sciences, University of Manchester, Manchester, UK; 9 Division of Cardiovascular Sciences, Faculty of Biology Medicine and Health, School of Medical Sciences, University of Manchester, Manchester, UK; 10 Division of Neuroscience and Experimental Psychology, Faculty of Biology, Medicine and Health, School of Biological Sciences, University of Manchester, Manchester, UK

**Keywords:** Predictive markers, CNS cancer, Cancer microenvironment, CNS cancer, Cancer imaging, Tumour biomarkers, Translational research

## Abstract

Stereotactic radiosurgery (SRS) is an established, effective therapy against vestibular schwannoma (VS). The mechanisms of tumour response are, however, unknown and in this study we sought to evaluate changes in the irradiated VS tumour microenvironment through a multinuclear MRI approach. Five patients with growing sporadic VS underwent a multi-timepoint comprehensive MRI protocol, which included diffusion tensor imaging (DTI), dynamic contrast-enhanced (DCE) MRI and a spiral ^23^Na-MRI acquisition for total sodium concentration (TSC) quantification. Post-treatment voxelwise changes in TSC, DTI metrics and DCE-MRI derived microvascular biomarkers (K^trans^, v_e_ and v_p_) were evaluated and compared against pre-treatment values. Changes in tumour TSC and microvascular parameters were observable as early as 2 weeks post-treatment, preceding changes in structural imaging. At 6 months post-treatment there were significant voxelwise increases in tumour TSC (*p* < 0.001) and mean diffusivity (*p* < 0.001, repeated-measures ANOVA) with marked decreases in tumour microvascular parameters (*p* < 0.001, repeated-measures ANOVA). This study presents the first in vivo evaluation of alterations in the VS tumour microenvironment following SRS, demonstrating that changes in tumour sodium homeostasis and microvascular parameters can be imaged as early as 2 weeks following treatment. Future studies should seek to investigate these clinically relevant MRI metrics as early biomarkers of SRS response.

## Introduction

Sporadic vestibular schwannomas (VS) are benign tumours arising from the Schwann cells lining the vestibulocochlear or eighth cranial nerve within the cerebellopontine angle (CPA)^1–3^. With an incidence of 15–20 per million per year, VS account for nearly 8% of all intracranial tumours and although histologically benign they can cause considerable morbidity due to both their anatomical location and unpredictable, sometimes rapid growth^1,3,4^. Traditionally the mainstay of management for growing tumours has been microsurgical resection but stereotactic radiosurgery (SRS) is also an effective treatment alternative in selected patients^5–7^. SRS offers several distinct advantages over surgical resection not least of which is the shorter recovery post-treatment and the potentially lower risk of facial nerve injury^6,7^. There are nonetheless well recognized challenges in the use of SRS as a therapy in these tumours. Unlike surgery where total or near total macroscopic tumour resection is often achieved, SRS does not acutely reduce tumour volume but rather attenuates tumour growth over a variable period of many months to years^8,9^. The timeline to volume reduction in any given tumour is therefore unpredictable and following treatment there is a well recognized 10% of VS that demonstrate continued tumour growth^8,9^.

At present, there is no way to predict response to SRS either prior to or immediately following treatment. Assessing success or failure of treatment is further complicated by the fact that reactive swelling and radiological appearance of tumour enlargement is commonly seen for up to 3 years after irradiation, following which shrinkage may be seen^8,9^. There is currently no way of determining whether this continued growth is treatment failure or reactive swelling^6,9^. The inability to differentiate between these two radically different responses to treatment means that cases of treatment failure are not recognized until after 3 years of continued growth, with the consequent surgical challenges of dealing with what is likely to be a significantly larger tumour with potential radiotherapy induced adherence to the adjacent cranial nerves and brain stem^8,10^.

Fundamental to these clinical challenges is our incomplete understanding of how and why these tumours respond to radiotherapy in the way they do^11,12^. Radiotherapy as a treatment preferentially targets rapidly dividing cells but studies to date have shown that schwannoma cell proliferation rates in VS are low and poorly associated with tumour growth^13–15^. Indeed recent research has demonstrated that, within growing tumours, infiltrating macrophages rather than neoplastic Schwann cells account for the majority of proliferating cells^1,3,16^. To better predict and monitor treatment responses in VS following SRS we, therefore, need a greater understanding of how the microenvironment in these tumours changes following treatment.

In other tumours treated with SRS such as cerebral metastases, advanced diffusion and perfusion MR imaging techniques have demonstrated utility in monitoring treatment responses. In such studies early reductions in tumour cytoarchitecture and reductions in perfusion MRI parameters such as the fractional plasma volume, v_p_, and the transfer constant, K^trans^ (a hybrid parameter reflecting tissue blood flow, vessel surface area, and vessel permeability), have been demonstrated following therapy^17–22^. There has been a paucity of such studies in VS, however. One advanced MR imaging technique which has received increased attention as a marker of treatment response in neuro-oncology is sodium (^23^Na) MRI^23–25^. Quantifying changes in total tissue sodium concentration (TSC) provides information on cellular physiology and tumour microstructure not obtainable with conventional ^1^H MRI and ^23^Na-MRI has been shown to be sensitive to therapy-induced changes in both systemic and central nervous system tumours^23–28^. The goal of the present study was to therefore use both ^23^Na-MRI and a comprehensive ^1^H-MRI protocol including diffusion MRI and dynamic contrast-enhanced (DCE) MRI to evaluate cellular, microstructural and microvascular changes in VS following SRS.

## Methods

### Patient population and treatment

Between February and June 2019 five patients with sporadic VS were recruited via the Skull Base Unit multidisciplinary team (MDT) meeting at our institution. All patients had undergone routine MR follow-up as part of their clinical care and all tumours were classified as growing by the multidisciplinary team meeting. Between March and June 2019, all five patients underwent single-fraction stereotactic radiosurgery (SRS) as the primary treatment modality. All patients were treated with frameless linear accelerator-based SRS using the Novalis TX and ExacTrac treatment systems (Varian Medical Systems, Palo Alto, CA, USA) equipped with 6MV SRS mode, HD120 multi-leaf collimator (MLC). An aquaplast mask was used for non-invasive immobilization. For treatment planning patients underwent stereotactic MRI at 1.5 T (General Electric Medical Systems, Milwaukee, WI, USA) and MRI images were then fused to high-resolution CT obtained at the time of simulation to assist in treatment volume design. Dosimetric planning was performed using the Pinnacle planning system and all patients received 12 Gy in a single fraction. Ethical approval was in place for the study from the National Research Ethics Service Greater Manchester North-West research ethics committee (REC reference: 18/NW/0094) and all patients provided written informed consent for study participation. All research was performed in accordance with local policies and guidelines and in accordance with the Declaration of Helsinki.

### MRI acquisition

In addition to standard pre-treatment clinical MR sequences, patients underwent imaging with a comprehensive research MRI protocol at the following timepoints: pre-treatment (4 patients), 2 weeks post-treatment (5 patients), 8 weeks post-treatment (4 patients) and 6 months post-treatment (4 patients). Two patients underwent imaging at four timepoints (pre-SRS, 2 weeks post-treatment, 8 weeks post-treatment, and 6 months post-treatment) and three patients were imaged at three timepoint. Details of when each patient underwent the research MRI protocol are shown in Table 1. For all patients, MRI data were acquired on a 3 Tesla whole-body scanner (Philips Achieva, Philips Medical System, NL). High spatial resolution 3D T1W (T1W) gradient-echo images with full brain coverage (TE 3.2 ms, TR 8.6 ms, voxel size 1 × 1 × 1 mm) both before and after contrast agent (gadoterate meglumine; Dotarem, Guerbet S.A.) administration was obtained for tumour delineation. A focused high-spatial resolution T2W-DRIVE (T2-weighted fast imaging with driven equilibrium radiofrequency reset pulse) acquisition through the CPA was also obtained (TE 237 ms, TR 2000 ms, voxel size 0.23 × 0.23 × 0.5 mm). Diffusion-tensor imaging (DTI) was performed using 15 directions and two b-values (0 and 800 s/mm^2^) and acquisition parameters were as follows: TE 74 ms, TR 6056 ms, voxel size 1.75 × 1.75 × 2 mm.

**Table 1:** Clinical features, delivered radiation dose and details of when each patient underwent the research MRI protocol.

Patient	1	2	3	4	5
Age	70.2	71.2	69.9	73.7	79.1
Gender	F	F	F	M	F
Tumour laterality	Right	Left	Right	Right	Left
Pre-treatment tumour volume (cm^3^)	0.85	1.3	1.03	1.59	1.2
Tumour Koos grade	III	III	III	III	III
Prescription dose (gy)	12	12	12	12	12
Gardner-Robertson (GR) hearing classification	II (Serviceable)	IV (Poor)	I (Good)	III (Non-serviceable)	II (Serviceable)
Mean dose-gross total volume (cGy)	1467.7	1448.2	1471.1	1467	1434.9
Mean Dose- planned target volume (cGY)	1423.5	1400.5	1436.2	1434.9	1427.1
Pre-treatment Imaging	Y	X	Y	Y	Y
2 weeks post-treatment imaging	Y	Y	Y	Y	Y
8 weeks post-treatment imaging	Y	Y	X	Y	Y
6 months post-treatment imaging	X	Y	Y	Y	Y

^a^Age at time of SRS treatment.

### DCE-MRI protocol

DCE-MRI data were collected at three timepoints (pre-treatment, 2 weeks, and 6 months post-treatment) using a previously described dual-injection, dual temporal resolution (DTR) technique^29^. A macrocyclic gadolinium-based contrast agent (GBCA, gadoterate meglumine; Dotarem, Guerbet S.A.) was administered by power injector as an intravenous bolus at a rate of 3 ml/s, followed by a chaser of 20 ml of 0.9% saline administered at the same rate. For the first part of this DTR technique, a high temporal but low spatial resolution sequence with a low dose of GBCA (fixed volume of 3 ml) was performed (LDHT-DCE). The LDHT-DCE series used a 3D GRE sequence with a flip angle of 16°, a field-of-view of 240 × 240 mm, image matrix of 96 × 96 × 22 voxels, and temporal resolution (Δ*t*) of 1.3 s (n = 300). Subsequently, a full GBCA dose (0.1 mmol/kg), high spatial but low temporal resolution (FDHS DCE) acquisition (voxel size of 1 × 1 × 2 mm; matrix size of 240 × 240 × 70, Δ*t* = 10.1 s, n = 60) was performed to provide high spatial resolution data. Variable flip angle (VFA; *α* = 2°, 6°, 12° and 16°) acquisitions were performed prior to the LDHT and FDHS DCE series for native longitudinal relaxation rate (R1_N_) mapping.

### ^23^Na- MRI protocol

Prior to the above ^1^H sequences a ^23^Na-MRI acquisition for TSC estimation was performed using a separate dual tuned ^1^H/^23^Na birdcage head coil (RAPID Biomedical, Rimpar, Germany). The total acquisition time was approximately ~ 14 min. To allow for quantification of TSC in both tumour and normal appearing brain, two sodium calibration phantoms made from a stock of 60 mM and 120 mM sodium chloride (NaCl) in water with 4% agarose was used and placed beside the ear defenders/headphones worn by the subjects to include them in the imaging field-of-view (FOV)^23,30^. The spiral ^23^Na-MRI images were reconstructed on the scanner and the TSC maps were subsequently calculated offline using MATLAB 2015a (The MathWorks, Inc., Natick, MA, USA)^31^. Further details on the ^23^Na- MRI protocol and analysis can be found in Supplementary methods.

### ^1^H-MRI analysis

DTI data were processed using a standard multistep procedure and the FSL 4.1 Diffusion Toolbox (http://www.fmrib.ox.ac.uk/fsl/)^32,33^. After correction for misregistration between successive images due to Eddy currents and/or patient movement, brain extraction was undertaken and voxelwise maps of mean diffusivity (MD) and fractional anisotropy (FA) were generated using the “DTIFIT” tool within FSL^16,32,33^.

Voxelwise maps of the microvascular kinetic parameters K^trans^, *v*_p_ and v_e_ (the fractional volume of extravascular extracellular space) were derived from the DCE-MRI datasets using the extended Tofts model (ETM)^22^. To permit high spatial resolution assessment of changes in tumour microvascular parameters following SRS treatment, a previously tissue-validated DCE-MRI analysis technique, termed LEGATOS (**LE**vel and rescale the **Ga**dolinium contrast concentrations curves of high-temporal **TO** high **S**patial DCE-MRI) was adopted^34,35^. For vascular input function (VIF) estimation the dynamic MRI signal from voxels in the superior sagittal sinus (SSS) was used^36^. An example VIF used for the DCE-MRI analysis is shown in Supplementary Fig. S1 alongside an example fit to a tumour. Further details on the LEGATOS analysis technique and the derivation of the VIF for this analysis can be found in supplementary methods and included reference^35^.

### Image co-registration and segmentation

Longitudinal co-registration was performed for each participant for all scan visits. TSC maps, DCE-MRI microvascular kinetic parameter maps and diffusion parameter maps from each visit were co-registered to the pre-treatment dataset using a rigid affine co-registration within SPM12^37^ (SPM12, UCL, London, UK). In all cases the accuracy of co-registration was visually inspected prior to further analyses. To allow accurate comparison of longitudinal changes in imaging parameters (TSC, diffusion metrics and DCE-MRI derived microvascular parameters) in each tumour ROI and across each tumour voxel, individual tumours were manually delineated on the co-registered pre-treatment T1W post-contrast image using Analyze version 11 (Biomedical Imaging Resource, Mayo Clinic, Rochester, MN, USA) to create a tumour object mask. These object masks, defined on the pre-treatment T1W post-contrast image for each patient, were then applied to the co-registered TSC maps, DCE-MRI microvascular kinetic parameter maps and the diffusion parameter maps (MD, FA).

To evaluate TSC values in normal appearing brain, tissue masks of white matter (WM), grey matter (GM), and cerebrospinal fluid (CSF) were derived through automated segmentation of co-registered 3D T1W images (SPM12, UCL, London, UK). To reduce the effect of partial volume, only voxels with a tissue probability larger than 95% were included in the segmented GM, WM, and CSF masks^23^. To corroborate the TSC values obtained through automated segmentation a hand‐drawn region-of-interest (ROI) analysis was performed using Analyze version 11. ROIs were drawn within the normal appearing white matter of the centrum semiovale (CS) and within the CSF of the lateral ventricles for each patient.

To evaluate the repeatability of DCE-MRI derived microvascular parameters across each timepoint a reference ROI mask was manually delineated on muscle tissue (masseter/temporalis muscle) within the imaging FOV^38–40^. This muscle ROI mask was delineated on the co-registered pre-treatment T1W post-contrast image using Analyze version 11 (Biomedical Imaging Resource, Mayo Clinic, Rochester, MN, USA) and then applied to the co-registered DCE-MRI microvascular kinetic parameter maps so that longitudinal temporal changes in muscle microvascular parameters could be assessed.

### Tumour volume estimation

For evaluation of tumour volume changes during treatment individual tumour volumes at each timepoint were determined through semi-automatic segmentation using the BrainLab iPlan software (BrainLAB, Feldkirchen, Germany). In addition to the study MRI scans, volumetric measurements of tumour size were also made for the preceding clinical scan so that the pre-treatment volumetric growth rate for each individual VS could be calculated^3,16^. Acquired high spatial resolution T2W images (i.e., T2W-DRIVE) was used for all segmentations and corroborated where available against tumour volumes measured on T1W post–contrast sequences. The results of all segmentations were reviewed and where necessary, edited by an experienced neuroradiologist.

### Statistical analysis

Stata version 11 and the SPSS statistical software package (version 25, IBM Corp.) were used for all statistical analyses. The test–retest coefficient of variation (CoV) was used for testing the intra-subject repeatability of GM, WM, and CSF TSC values across each imaging timepoint and the repeatability of DCE-MRI derived microvascular parameters within the reference muscle ROI. For each subject, **i**, the CoV is the SD, **σ**_**i**_, for all measurements on that subject, divided by the mean, **μ**_**i**_, for the subject. The global test–retest CoV for a group of **N** subjects was defined as $\sqrt{\sum(\sigma/\mu )^2/N}$^41,42^. The average measures intraclass correlation coefficient of TSC values from each atlas (GM, WM, CSF) and ROI defined region (CS, CSF lateral ventricle) was also calculated using an absolute-agreement, 2-way mixed-effects model^43^.

Voxelwise temporal changes in TSC, diffusion metrics, and DCE-MRI derived microvascular kinetic parameters (K^trans^, v_e_ and v_p_) within each tumour voxel were evaluated using a repeated-measures ANOVA with Greenhouse–Geisser correction for non-sphericity. To account for missing timepoint data in three patients, temporal changes in voxelwise parameter values, mean whole tumour ROI values and tumour volume values across all patients studied were analysed using a repeated-measures mixed-effects model with imaging timepoint as a fixed-effects variable^44^. In both approaches post hoc analysis of pairwise comparisons between different timepoints were performed using the Bonferroni method.

The voxelwise relationship between TSC, diffusion metrics and DCE-MRI derived microvascular kinetic parameters (K^trans^, v_e_ and v_p_) at each timepoint was evaluated using scatterplot analysis and reported as Spearman’s rho. To evaluate the relationship between longitudinal changes in these metrics and changes in different tumoural cellular compartments, the percentage of voxels at each post-treatment timepoint demonstrating an increase in either the extracellular sodium population (defined as an increase in both TSC and MD or TSC and v_e_) or intracellular sodium population (defined as an increase in TSC with a decrease in MD or v_e_) relative to pre-treatment values was calculated and compared for non-equality using a one-sample test of proportions. The percentage of voxels displaying both increased TSC/MD and increased v_e_, K^trans^ or v_p_ was also derived and compared across each post-treatment timepoint.

## Results

### Patient population

Demographic details of the study patients are presented in Table 1. Median age at time of treatment was 71.2 years (Range 69.9–79.1 years). Four patients were female. In three patients the VS was right-sided. Median time between pre-treatment imaging and SRS was 4 days (range 1–6 days). The median time (range) to each post-treatment imaging timepoint was 14 days (13–16 days), 57 days (55–70 days), and 181 days (160–184 days) respectively. All tumours were Koos grade III^45^. Mean pre-treatment VS size was 1.16 ± 0.27 cm^3^ and mean pre-treatment growth rate was 0.59 ± 0.23 cm^3^/yr (Table 2). Mean tumour volume change at 2 weeks, 8 weeks and 6 months respectively was 0.01, 0.05, and 0.22 cm^3^ but these differences were not statistically significant (p > 0.05, repeated-measures mixed-effects model, Table 2). At time of clinical follow-up six weeks following SRS, hearing loss was reportedly worse in one patient (patient 1) and unchanged in 4 patients; one patient (patient 2) reported worsened left-sided facial sensory disturbance following treatment.

**Table 2:** Temporal changes in VS volume post-treatment.

Patient	Pre-treatment tumour growth rate (cm^3^/yr.)	Tumour volume, cm^3^			
		Pre	2 weeks	8 weeks	6 mon
1	0.24	0.76	0.72	0.70	
2	0.55	1.20	1.22	1.28	1.24
3	0.88	1.07	1.08		1.25
4	0.59	1.51	1.54	1.58	1.79
5	0.71	1.25	1.26	1.26	1.24
Mean ± SD	0.59 (0.23)	1.16 (0.27)	1.17 (0.28)	1.21 (0.37)	1.38 (0.27)
Mean difference from baseline, cm^3^ (95% CI)			0.01 (− 0.57, 0.59)	0.05 (− 0.57, 0.66)	0.22 (− 0.39, 0.84)
*P* value^a^			0.99	0.99	0.99

^a^Repeated measures mixed-effects model with post hoc pairwise comparison using the Bonferroni method.

Acquired high spatial resolution T2W images (i.e., T2W-DRIVE) was used for all volume segmentations across all timepoints.

### Total sodium concentration (TSC) in normal appearing brain

Mean total sodium concentration (TSC) in atlas defined GM and WM at each timepoint is shown in Supplementary Table S1. Mean pre-treatment TSC in atlas defined GM, WM, and CSF was 39.4 ± 3.11 mM, 35.4 ± 2.27 mM and 69.9 ± 3.39 mM respectively, in line with previously reported whole-brain literature values^23,25,46^. Use of a manual ROI based analysis displayed a comparable mean pre-treatment WM TSC of 35.8 ± 4.03 mM for centrum semiovale and a higher CSF TSC of 113 ± 4.71 mM within the lateral ventricle, reflecting the reduced partial volume effects on measured CSF TSC using this approach (supplementary Table S2).

Intra-subject variance of TSC values in normal appearing brain across each timepoint was low with mean CoV values of 9.0%, 10.2%, and 4.25% for atlas defined GM, WM, and CSF respectively (Supplementary Table S3). Intra-observer repeatability across all regions was good (ICC > 0.65) with both atlas-defined and ROI-based CSF values demonstrating excellent repeatability (ICC 0.91–0.94). There was no correlation between serum Na^+^ concentration and TSC values in either normal appearing brain or tumour (Spearman’s rho, *p* > 0.05).

### Sodium bioscales demonstrate early post-SRS changes in VS

**Figure 1:**
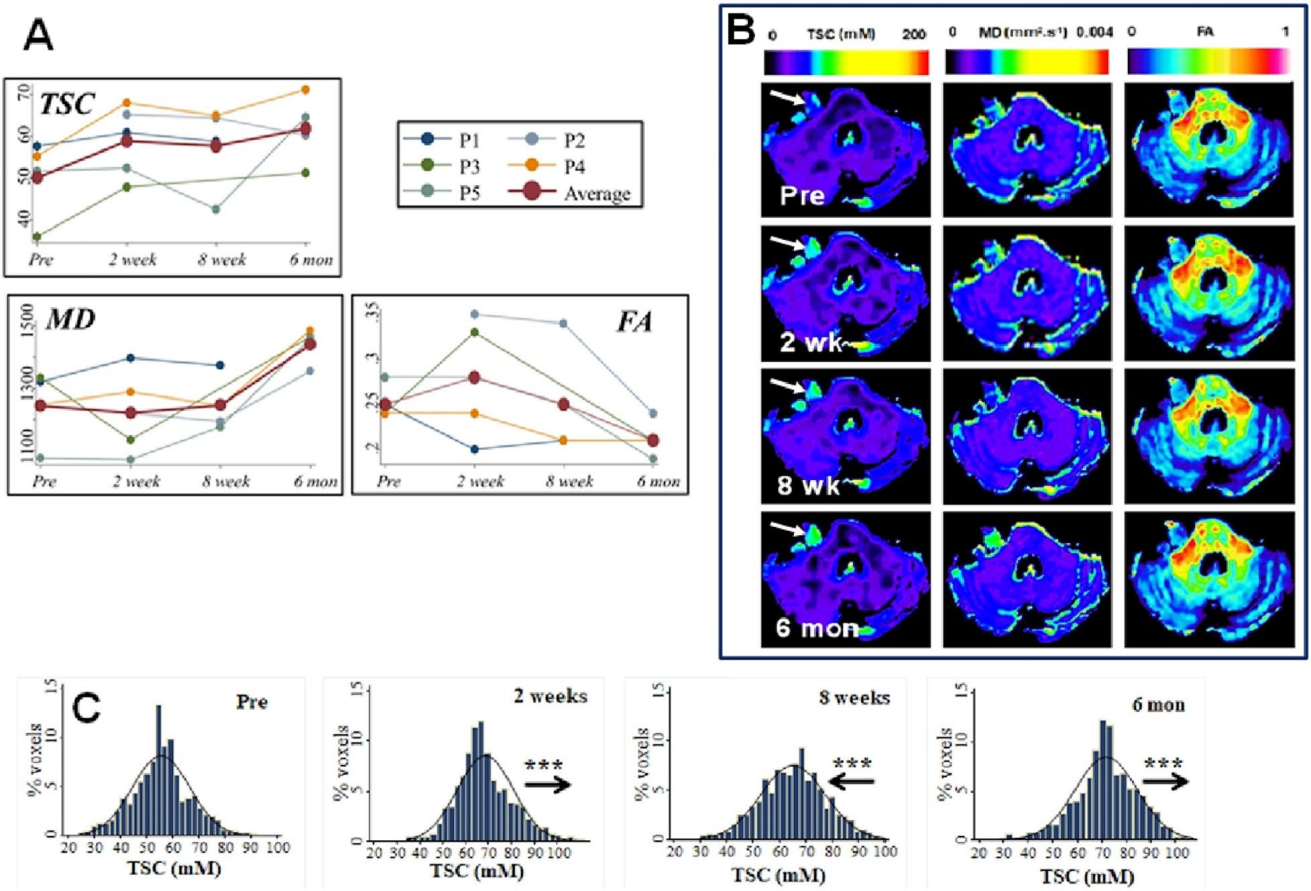
Post-SRS changes in tumour total sodium concentration (TSC) and diffusion metrics (MD, FA). (**A**) Line-graphs demonstrating temporal trend in mean tumour TSC (mM), MD (mm^2^ s^−1^ × 10^−6^) and FA (no units) for five VS. Average trend (red line) across all tumours also shown. (**B**) Representative TSC and diffusion metric maps from the right-sided growing VS shown in Panel A (patient 4, P4). From left to right: co-registered voxelwise maps of TSC, MD, and FA. Note the observable increase in TSC at 2 weeks, 8 weeks and 6 months post-SRS compared to pre-treatment values within the tumour (*arrow*) and the comparatively later visible increase in MD and reduction in tumoural FA observable at 6 months post-SRS. (**C**) Histogram of voxelwise TSC values within the tumour shown in panel B (patient 4, P4). From left to right: Pre-treatment, 2 weeks post-treatment, 8 weeks post-treatment, and 6 months post-treatment. Note the rightward shift in voxelwise TSC values at 2 weeks, 8 weeks, and 6 months post-treatment compared to pre-treatment values. ***p < 0.001 for comparison with pre-treatment TSC value, repeated-measures ANOVA with post hoc (Bonferroni) analysis of pairwise comparisons. Arrows show movement in histogram relative to preceding imaging timepoint. *FA* Fractional anisotropy (no units); *MD* Mean diffusivity (mm^2^ s^−1^ × 10^−6^); *TSC* Total sodium concentration (mM).

Mean pre-treatment TSC across the four VS with available imaging was 50.1 ± 26.6 mM (Supplementary table S4) and was significantly higher than WM TSC values (*p* = 0.02, ANOVA). Changes in total sodium concentration were evident as early as 2 weeks post-treatment and preceded changes in both structural imaging and diffusion parameter maps (Fig. 1, Supplementary Fig. S2,S3). As shown in Table 3 across all four tumours with pre-treatment imaging there was a significant voxelwise increase in TSC at 2 weeks (*p* < 0.001) and 6 months (*p* < 0.001) post-SRS compared to pre-treatment values (repeated-measures ANOVA).

**Table 3:** Voxelwise temporal changes in tumour total sodium concentration (TSC), mean diffusivity (MD) and fractional anisotropy (FA).

Patient	Voxelwise Δ TSC (mM) Mean difference (95% CI)			Voxelwise Δ MD (mm^2^ s^−1^ × 10^–6^) Mean difference (95% CI)			Voxelwise Δ FA Mean difference (95% CI)		
	Pre/2 weeks	Pre/8 weeks	Pre/6 months	Pre/2 weeks	Pre/8 weeks	Pre/6 months	Pre/2 weeks	Pre/8 weeks	Pre/6 months
1	3.62 (2.92,4.32)***	1.53 (1.13, 1.92)***		76.6 (53.5, 99.8)***	52.9 (22.0, 83.8)***		− 0.05 (− 0.06, − 0.04)***	− 0.03 (− 0.05, − 0.02)***	
2^a^		− 0.84 (− 1.27 − 0.40)***	− 5.07 (− 5.65,,− 4.50)***		− 25.8 (− 50.5,− 1.01)*	118.31 (85.5, 151.1)***		− 0.02 (− 0.03,− 0.005)**	− 0.11 (− 0.13, − 0.10)***
3	12.3 (11.6, 12.9)***		16.0 (15.3, 16.6)***	− 176 (− 206,− 144)***		171 (137, 203)***	0.08 (0.07,0.09)***		− 0.03 (− 0.04, − 0.02)***
4	12.7 (12.4, 13.1)***	9.65 (9.24, 10.1)***	16.0 (15.6, 16.4)***	32.3 (17.2, 47.3)***	− 3.06 (− 14.5, 8.40)	233 (216, 251)***	− 0.001 (− 0.006, 0.005)	− 0.03 (− 0.03, 0.02)***	− 0.06 (− 0.06, − 0.05)***
5	0.94 (0.46, 1.41)***	− 9.03 (− 9.85, − 8.20)***	13.1 (12.6, 13.7)***	− 3.75 (− 19.6, 12.1)	90.8 (76.8, 105)***	401 (371, 430)***	0.002 (− 0.006, 0.01)	− 0.03 (− 0.04, − 0.02)***	− 0.09 (− 0.10, − 0.08***
All	8.30 (7.91, 8.67)***	3.03 (2.54, 3.52)***	12.6 (12.2, 13.0)***	0.16 (− 11.9, 12.3)	22.5 (10.1,34.9)***	263 (249, 276)***	0.01 (0.01, 0.02)***	− 0.01 (− 0.02, − 0.007)***	− 0.06 (− 0.07, − 0.05)***

Statistically significant values (*p* ≤ 0.05) are shown in bold

*P* value calculated using repeated measures ANOVA or repeated measures mixed-effects model. Post hoc analysis of pairwise comparisons between different timepoints was performed using the Bonferroni method.

*** ≤ 0.001; ** ≤ 0.01; * ≤ 0.05.

^a^Difference from 2 week post-treatment timepoint shown as no pre-treatment imaging dataset.

At 8 weeks there was heterogeneity in tumour response with two tumours demonstrating a significant voxelwise increase in TSC (Patient 1 and 4, *p* < 0.001, Fig. 1) and 1 tumour demonstrating a significant decrease in TSC compared to pre-treatment values (patient 5, Supplementary Fig. S2). Whole tumour ROI analysis demonstrated that compared to pre-treatment values there were statistically significant increases in both absolute mean tumour TSC and the tumour-to-CSF TSC ratio at 6 months post-treatment (*p* ≤ 0.02, repeated-measures mixed-effects model, Supplementary Table S4).

### Temporal trends in tumour diffusion metrics (MD, FA) at 8 weeks and 6 months post-treatment mirror changes in tumour TSC

In the four VS studied there was a significant voxelwise reduction in FA at 8 weeks (*p* < 0.001) and 6 months post-treatment (*p* < 0.001, repeated-measures mixed-effects model, Table 3). In contrast to early (2 week) post-treatment TSC changes there was considerable heterogeneity in MD and FA values with tumours displaying an increase (patient 1 and 4, *p* < 0.001, Fig. 1) or decrease in voxelwise MD values compared to pre-treatment values (patient 3, *p* < 0.001, supplementary Fig. S3). In corroboration of the voxelwise changes, whole tumour ROI analysis (Supplementary Table S4) demonstrated that at 6 months post-treatment there were significant increases in tumour MD (*p* < 0.001) and decreases in mean tumour FA (p = 0.003, repeated-measures mixed-effects model).

### Temporal changes in VS microvascular parameters are visible as early as 2 weeks post-SRS

There was no significant change in the v_e_, K^trans^ or v_p_ values of the reference muscle ROI at any timepoint (*p* > 0.05, repeated measures mixed-effects model, supplementary table S5). The intra-subject variability of v_e_, K^trans^ and v_p_ values for muscle across each imaging timepoint was low with mean CoV values of 11.8%, 23.2% and 27.2% respectively (Supplementary Table S5).

**Figure 2:**
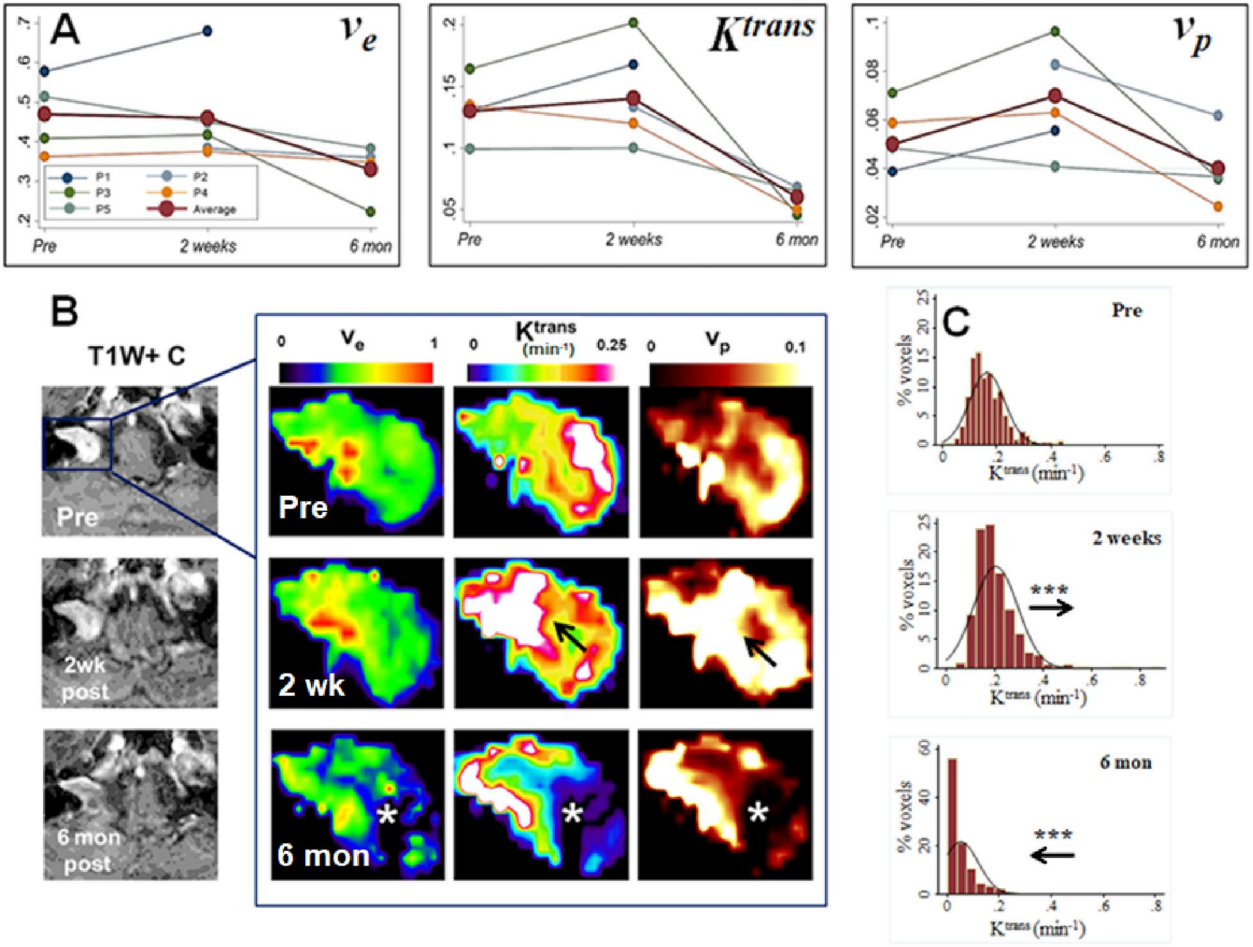
Post-SRS changes in tumour microvascular parameters. (**A**) Line-graphs demonstrating temporal trend in mean tumour v_e_ (no units), K^trans^ (min^−1^) and v_p_ (no units) for five VS. Average trend (red line) across all tumours also shown. (**B**) Representative parameter maps from the right-sided growing VS shown in Panel A (patient 3, P3). From left to right: T1W post-contrast, co-registered voxelwise maps of v_e_, K^trans^, and tumour v_p_. Note the early increase in tumour K^trans^ and v_p_ at 2 weeks post-treatment (*arrow*) followed by marked reductions in v_e_, K^trans^, and v_p_ within the cerebellopontine angle portion of the tumour at 6 months post-SRS (*). (**C**) Histogram of voxelwise K^trans^ values within the tumour pre-treatment (top), 2 weeks post-treatment (middle) and 6 months post-treatment (bottom). ***p < 0.001 for comparison with pre-treatment TSC values, repeated-measures ANOVA with post hoc (Bonferroni) analysis of pairwise comparison. Arrows show movement in histogram relative to preceding imaging timepoint.

In Table 4 voxelwise temporal changes in tumour DCE-MRI derived microvascular parameters are shown. There were significant voxelwise decreases in v_e_ (*p* < 0.001), K^trans^ (*p* < 0.001) and v_p_ (*p* < 0.001) at 6 months post-treatment compared with pre-treatment values (repeated-measures ANOVA). This decrease was clearly visualized on acquired parametric maps, occurred predominantly within the CPA portion of the tumour, and was associated with a reduction in central contrast enhancement on post-contrast T1W imaging (Fig. 2, Supplementary Fig. S4,S5). Changes in tumour microvascular parameters at the early 2 week post-treatment timepoint were more heterogenous (Fig. 2). Two patients demonstrated increases in v_e_, K^trans^ and v_p_ at 2 weeks post-treatment (patient 1 and 3, Fig. 2), one patient demonstrated increases in v_e_ and v_p_ only (patient 4, Supplementary Fig. S4) and one patient demonstrated significant decreases in all three microvascular parameters at 2 weeks (*p* < 0.001, repeated-measures ANOVA, patient 5, Supplementary Fig. S5). Temporal changes in whole tumour ROI DCE-MRI derived microvascular parameters are shown in Supplementary Table S6.

**Table 4:** Voxelwise temporal changes in tumour DCE-MRI derived microvascular parameters.

Patient	Voxelwise Δv_e_ (no units) Mean difference (95% CI)		Voxelwise Δ K^trans^ (min^−1^) Mean difference (95% CI)		Voxelwise Δ v_p_ (no units) Mean difference (95% CI)	
	Pre/2 weeks	Pre/6 months	Pre/2 weeks	Pre/6 months	Pre/2 weeks	Pre/6 months
1	0.11 (0.09,0.13)***		0.04 (0.03, 0.04)***		0.009 (0.005,0.14)***	
2^a^		− 0.02 (− 0.04, 0.002)		− 0.06 (− 0.07,− 0.06)***		− 0.02 (− 0.02, − 0.01)***
3	0.004 (− 0.008, 0.16)	− 0.20 (− 0.22, − 0.18)***	0.04 (0.03,0.05)***	− 0.12 (− 0.13, − 0.10)***	0.02 (0.02, 0.03)***	− 0.03 (− 0.04,− 0.03)***
4	0.01 (0.008,0.02)***	− 0.02 (− 0.03,− 0.005)**	− 0.02 (− 0.02, − 0.01)***	− 0.08 (− 0.09,− 0.08)***	0.004 (0.003, 0.006)***	− 0.03 (− 0.04, − 0.03)***
5	− 0.07 (− 0.09, − 0.06)***	− 0.13 (− 0.16, − 0.11)***	**− 0.001 (− 0.004, 0.003)**	− 0.03 (− 0.03, − 0.02)***	− 0.007 (− 0.01, − 0.005)***	− 0.004 (− 0.007, 0.00)*
All	− 0.004 (− 0.01, 0.004)	− 0.09 (− 0.10, − 0.08)***	0.009 (0.005, 0.01)***	− 0.07 (− 0.074,− 0.067)***	0.008 (0.007, 0.01)***	− 0.02 (− 0.022, − 0.018)***

Statistically significant values (*p* ≤ 0.05) are shown in bold

*P* value calculated using repeated measures ANOVA or repeated measures mixed-effects model. Post hoc analysis of pairwise comparisons between different timepoints was performed using the Bonferroni method. ***≤ 0.001; **≤ 0.01; *≤ 0.05.

^a^Difference between post-treatment timepoints shown as no pre-treatment imaging dataset.

### Voxelwise correlation between TSC, diffusion metrics and microvascular parameters

With the exception of one tumour (patient 2) all VS demonstrated a significant (p ≤ 0.05) positive voxelwise correlation between TSC and mean diffusivity at each timepoint and a negative correlation between TSC and FA (Supplementary Table S7). The correlation between TSC and mean diffusivity values across all tumour voxels was higher at 2 weeks (rho = 0.51, *p* < 0.001), 8 weeks (rho = 0.44, *p* < 0.001) and 6 months post-treatment (rho = 0.45, *p* < 0.001) compared to pre-treatment values (rho = 0.41, *p* < 0.001).

Across all tumour voxels there was a weak negative correlation of pre-treatment voxelwise TSC with v_e_ (rho = -0.06, *p* < 0.001), K^trans^ (rho = -0.04, *p* < 0.001) and v_p_ values (rho = -0.27, *p* < 0.001). At 6 months post-treatment there was a negative correlation of TSC with both K^trans^ (rho = -0.24, *p* < 0.001) and v_p_ (rho = -0.24, *p* < 0.001), with no correlation observed between TSC and v_e_ (rho = -0.002, p = 0.92). The voxelwise correlation between tumour diffusion metrics (MD, FA) and tumour DCE-MRI derived microvascular parameters (v_e_, K^trans^ and v_p_) is shown in Supplementary Table S8. Pre-treatment across all tumour voxels there was an inverse correlation between MD and v_e_ values (rho = -0.38, *p* < 0.001), and at 6 months post-treatment MD correlated inversely with all three microvascular parameters (*p* < 0.001).

### Post-SRS increases in tumour TSC reflect increases in the extravascular-extracellular space and correspond with regions of reduced tumour vascularity at 6 months post-treatment

**Figure 3:**
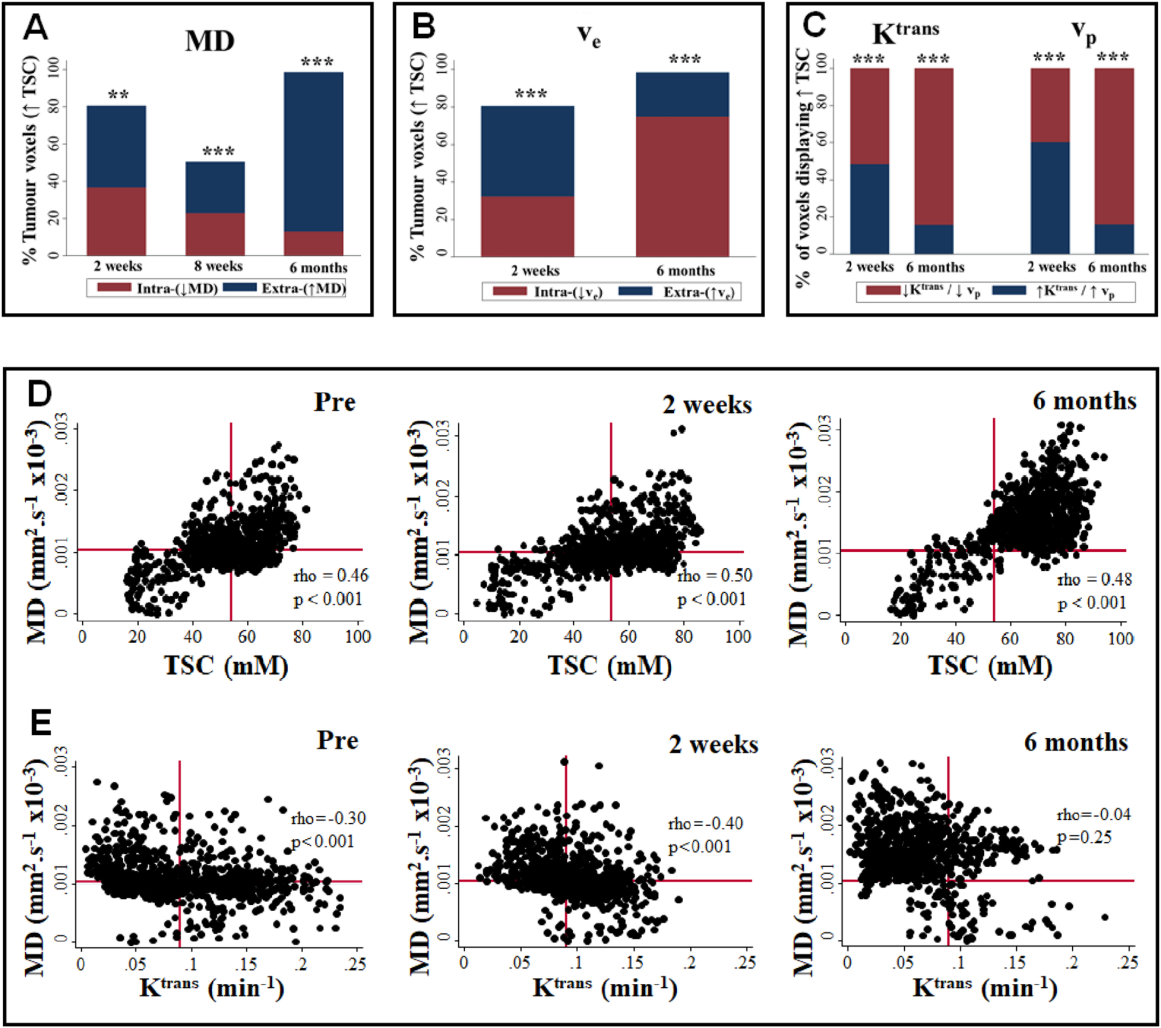
Temporal changes in extracellular and intracellular total sodium population and DCE-MRI derived microvascular parameters. (**A**) Bar-chart showing percentage of all tumour voxels demonstrating increased TSC and an increase (↑TSC: ↑MD) or decrease in MD (↑TSC:↓MD) at 2 weeks, 8 weeks, and 6 months post-treatment. Note the increase in the percentage of tumour voxels displaying ↑TSC: ↑MD at 6 months post-treatment. (**B**) Bar-chart showing percentage of all tumour voxels demonstrating increased TSC and an increase (↑TSC: ↑v_e_) or decrease in v_e_ (↑TSC:↓v_e_) at 2 weeks and 6 months post-treatment. Note the increase in the percentage of tumour voxels displaying ↑TSC but ↓v_e_ at 6 months post-treatment. *P* value shown is for comparison of all tumour voxels displaying an increase in either the extracellular (↑TSC:↑MD or ↑TSC:↑v_e_) or intracellular (↑TSC:↓MD or ↑TSC:↓v_e_ ) sodium population (one-sample test of proportions **P < 0.01 ***P < 0.001). (**C**) Bar-chart showing percentage of tumour voxels with increased TSC (↑TSC) that display an increase (↑) or decrease (↓) in K^trans^ and v_p_ at 2 weeks and 6 months post-treatment. Note the increase in the percentage of tumour voxels displaying ↓K^trans^ and ↓v_p_ at 6 months post-treatment. *P* value shown is for comparison of all tumour voxels displaying either an increase or decrease in K^trans^ and v_p_ respectively (one-sample test of proportions **P < 0.01 ***P < 0.001). (**D**) Voxelwise scatterplot comparison of tumour TSC values and mean diffusivity (MD) estimates shown for patient 5 at different timepoints. Horizontal and vertical red bar indicates the 50% percentile for the pre-treatment voxelwise MD and TSC estimates respectively. (**E**) Voxelwise scatterplot comparison of tumour mean diffusivity (MD) values and K^trans^ estimates shown for patient 5 at different timepoints. Horizontal and vertical red bar indicates the 50% percentile for the pre-treatment voxelwise MD and K^trans^ estimates respectively. Note the increase in the percentage of tumour voxels displaying ↑TSC and ↑MD but ↓K^trans^ at 6 months post-treatment. Spearman’s rho reported on scatterplots.

Figure 3A/B and Supplementary Table S9 shows the percentage of tumour voxels at each timepoint displaying increases in TSC, mean diffusivity and v_e_ compared to pre-treatment. The percentage of all voxels displaying an increase in TSC at 2 weeks, 8 weeks and 6 months following treatment was 80.9%, 61.5%, and 98.5% respectively. At 2 weeks post-treatment 43.1% of all tumour voxels displayed an increase in the extracellular sodium population (^↑23^Na: ↑MD). A significantly lower number of voxels (37.8 vs 43.1%, *p* < 0.001, one-sample test of proportions) demonstrated an increase in the intracellular sodium population (^↑23^Na: ↓MD). Voxelwise v_e_ values corroborated this with 50.3% and 30.6% of voxels demonstrating an increase in extracellular (^↑23^Na: ↑v_e_) and intracellular (^↑23^Na: ↓v_e_) sodium population respectively (*p* < 0.001, one-sample test of proportions).

Between 2 and 8 weeks post-treatment, the percentage of voxels demonstrating an increase in TSC relative to pre-treatment values decreased, with 34.8% and 26.7% of all voxels demonstrating increases in the extracellular and intracellular sodium population respectively when compared to pre-treatment values. At 6 months post-treatment, however, 98.5% of all tumour voxels demonstrated increases in TSC compared to pre-treatment with the large majority (83.5% vs 15.0%, *p* < 0.001, one-sample test of proportions) demonstrating increases in extracellular (^↑23^Na: ↑MD) rather than intracellular (^↑23^Na: ↓MD) sodium.

As shown in Supplementary Table S10 and Fig. 3C, the percentage of voxels with increased TSC or MD displaying increased K^trans^ decreased from ~ 50% at 2 weeks post-treatment to only ~ 16% at 6 months post-treatment. The v_e_ and v_p_ values paralleled these changes with ~ 76% and 83 – 85% of all tumour voxels displaying decreases in v_e_ and v_p_ respectively at 6 months, despite increases in MD and TSC. In Fig. 3D/E voxelwise scatterplots of TSC, mean diffusivity (MD) and K^trans^ are shown for a representative patient (patient 5) and clearly demonstrate the increase in the percentage of tumour voxels displaying increased TSC and MD but decreased K^trans^ at 6 months post-treatment.

## Discussion

This study demonstrates that advanced MRI techniques such as ^23^Na-MRI and DCE-MRI permit the early interrogation of changes in the microstructure, microvasculature, and cellular physiology of VS undergoing SRS. In particular we demonstrate that increases in tumour TSC measured through ^23^Na-MRI, are detectable as early as 2 weeks following radiotherapy, preceding changes in macroscopic tumour structure and tumour diffusion metrics. Through a concomitant DCE-MRI acquisition we furthermore show that early changes in the VS microvasculature can be detected following SRS and that following an early post-treatment rise in tumour microvascular parameters such as K^trans^ and v_p_ in some VS, there is a marked decrease in these parameters at 6 months following treatment.

TSC has been previously investigated as a biomarker of tumour response to fractionated chemo/radiotherapy but to date there have been no studies in histologically benign tumours such as VS^23–25,27,28^. Thulborn et al. interrogated changes in TSC at the voxel level in patients with high grade glioma undergoing fractionated radiotherapy, demonstrating that voxelwise TSC values were responsive to radiation effects on a weekly time scale, with ‘responsive’ tumour regions displaying an early sharp increase in TSC following radiotherapy^24,25^. Huang et al. in a 7 T study of a cerebral metastasis undergoing SRS similarly demonstrated a dramatic increase in the ^23^Na-MRI signal intensity within the tumour at 48 h post-treatment, an effect the authors postulated was due to radiotherapy-induced cellular apoptosis^47^.

In the present study we show that following radiotherapy, there is an early rise in VS sodium content with > 80% of tumour voxels demonstrating an increase in TSC at 2 weeks post-treatment. Whilst an increase in intracellular sodium secondary to radiation induced cellular apoptosis or disrupted cellular membrane ion transport may in part underlie this increase, the majority of voxels demonstrating increased TSC also displayed concomitant increases in mean diffusivity^28,48^, a biomarker thought to predominantly reflect extracellular diffusion. This indicates that the TSC rise observed in the majority of tumour voxels at 2 weeks is likely reflective of increases in the extravascular-extracellular space, a finding corroborated by the comparable voxel percentage demonstrating an increase in both v_e_ and TSC at this timepoint. Necrosis of radiosensitive tumour cell populations could in part explain this early rise in TSC, but the demonstrated rise in the DCE-MRI derived metric K^trans^ in some tumours suggest that increases in tumoural vascular permeability and interstitial oedema may also be a possible mechanism through which this occurs.

At 2 and 8 weeks post-treatment there was considerable heterogeneity across the VS studied with different tumours displaying increases and decreases in TSC and MD respectively., This may be explained by variations between individual tumours and tumour regions in the extent and timing of varying pathophysiological mechanisms in response to radiotherapy, although slight differences in the timing of imaging following SRS may also be a factor. Membrane ion transporter failure, cellular apoptosis and swelling could all act to increase intracellular sodium content and restrict water diffusion whilst simultaneously within the same tumour there is loss of barriers to water diffusion and increased extracellular sodium secondary to necrosis and interstitial tumoural oedema. To better clarify changes in water and sodium distribution following SRS, further studies should be undertaken which incorporate both multi-compartment diffusion MRI protocols^49^, and dedicated ^23^Na-MRI acquisitions for direct estimation of the intracellular sodium population^50^.

Following an early post-treatment rise in some tumours, we demonstrate that at 6 months post radiotherapy there is a marked decrease in the DCE-MRI derived microvascular parameters v_p_, K^trans^ and v_e_. Whilst there is a paucity of comparable studies in other histologically benign tumours, previous studies in cerebral metastases have demonstrated similar reductions in DCE-MRI derived microvascular parameters following SRS treatment^17,18^. It has been proposed that the radiation sensitivity of tumours to dose fractions of 10 Gy or more is governed by the sensitivity of tumour endothelial cells to apoptosis and in the early post-irradiation period endothelial damage and increased capillary membrane permeability has been reported^51,52^. Such an effect may underlie the early increases in K^trans^, v_e_ and TSC seen in some VS^52^. Conversely early increases in capillary membrane permeability and plasma extravasation may result in the observed early reduction in the measured ‘functional’ microvasculature in some tumours, as erythrocyte concentration and elevation of interstitial fluid pressure above the intravascular blood pressure leads to vascular stasis and vascular collapse in the smaller vessels^52^.

In malignant tumours, both direct cellular DNA damage leading to cellular necrosis/apoptosis and indirect mechanisms such as vascular damage induced hypoxia are thought to contribute to tumour cell death and macroscopic radioresponse^52–54^. Our demonstration that there are increases in TSC and MD and decreases in tumour vascularity at 6 months post-SRS, with a demonstrable cohort of voxels displaying low v_e_/K^trans^/v_p_ and high TSC/MD, suggest that similar mechanisms also occur in histologically benign tumours such as VS^11,12^. At low diffusion gradient b-values (*b* < 200 s/mm^2^), such as those used within the DTI acquisition in this study, increased microvascular perfusion can be misinterpreted as diffusion effects, resulting in increases in scalar diffusion metrics such as mean diffusivity^55^. Although it is possible that such effects may explain some of the observed increases in both MD and DCE-MRI derived microvascular parameters at 2 weeks post-treatment, given the generally low plasma volume fraction, v_p_ (< 10%) in these tumours and the very low number of voxels displaying increased K^trans^/v_p_ at 6 months post-treatment, it is unlikely that perfusion effects play a dominant role in explaining the increased post-treatment MD and TSC values observed. Further studies, however, that incorporate more advanced diffusion acquisitions, such as IVIM (intravoxel-incoherent motion) imaging, that allow specific assessment of the vascular contribution to the diffusion signal should be undertaken^56^.

As a treatment modality radiotherapy preferentially targets proliferating tumour cell populations in the M and G_2_ phase of the cell cycle^11,57^, and within growing VS tumour associated macrophages (TAM) rather than Schwann cells were previously found to account for the majority of Ki67^+^ proliferating cells^1,3,16^. Alongside Schwann cell death, apoptosis or necrosis of radiosensitive TAM populations may also contribute to the observed changes in TSC and MD seen^58^. In previous studies an intimate link between tumour vascularity and infiltration of VEGF-expressing TAMs in VS has been demonstrated and our findings raise the interesting possibility that the observed growth attenuation seen in responsive tumours following SRS is also a manifestation of reduced tumoural vascular supply and decreased infiltration of pro-tumourigenic TAMs into the tumour microenvironment^1,3,16^. Whilst the absence of resected tissue in SRS treated VS cohorts limits the ability to test these hypotheses directly future human imaging studies incorporating DCE-MRI and established PET tracers of inflammation in VS could be undertaken so that post-SRS changes in tumoural TAM density can be evaluated in vivo^1,3,16^.

The present study is the first demonstration that early changes in the cellular viability, microstructure and microvasculature of SRS treated VS can be detected in vivo using clinically applicable MRI techniques. A limitation of this pilot study, however, is that the number of study patients is low and a key question for future studies is the generalisability of the observed findings across the whole spectrum of VS undergoing radiotherapy. Within this study radiological follow-up of included patients was furthermore limited to only 6 months. Studies have shown that up to 63% of SRS treated patients can show transient tumour expansion or swelling after SRS and our volumetric tumour measurements support this, demonstrating a non-significant increase in VS volume at 6 months post-treatment^8,9^. Pollock et al. showed that 57% of tumours with transient swelling demonstrated eventual tumour regression, with many authors recommending at least two years of radiological follow-up before declaring treatment a failure^8,9^. Ultimately, the goal would be that these early imaging changes correlate to and predict the success or failure of radiotherapy. This will of course require future studies incorporating a larger population of VS and long-term imaging follow-up at 18 months and 3 years post-treatment to evaluate the ability of these early imaging biomarkers to predict later tumour response and differentiate true treatment failure from transient swelling.

## Conclusions

Through a pilot, multinuclear MRI study we present the first in vivo study of temporal changes in the microstructure, microvasculature, and cellular physiology of VS undergoing SRS treatment. We demonstrate that changes in sodium homeostasis and the tumour microvasculature occur as early as 2 weeks post-treatment and that by 6 months post-SRS there are demonstrable decreases in MRI markers of cellular density and the microvasculature in treated VS. Our study provides new insights into the post-SRS VS microenvironment and future studies should investigate these MRI metrics as early biomarkers of SRS response.

## Supplementary Information

Supplementary Information.

Supplementary Tables.

## Data Availability

The datasets generated during and/or analysed during the current study are available from the corresponding author on reasonable request.
