# Supplementary material for: Detection of early changes in the post-radiosurgery vestibular schwannoma microenvironment using multinuclear MRI
Source: Sci Rep. 2021 Aug 3;11:15712. doi: 10.1038/s41598-021-95022-6 (PMC8333359; doi:10.1038/s41598-021-95022-6)
Supplement: Supplementary file 1 — Supplementary Information. [file 41598_2021_95022_MOESM1_ESM.docx]

**Detection of early changes in the post-radiosurgery vestibular schwannoma microenvironment using multinuclear MRI**

**Daniel Lewis MRCS^1,2,3*^, Damien J McHugh PhD^4^, Ka-loh Li PhD^3^, Xiaoping Zhu PhD^3^, Catherine Mcbain MD^2,5^,** [**Simon K. Lloyd**](https://thejns.org/search?f_0=author&q_0=Simon+K.+Lloyd)**FRCS(ORL-HNS)^6^, Alan Jackson PhD^3^, Omar N Pathmanaban PhD^1,2,7^, Andrew T King FRCS (SN)^1,2,8^, David J Coope PhD^1,2,9*^**

# Supplementary methods

**Analysis of acquired DWI/DTI data**

Acquired DTI data for all patient cohorts was processed using the FSL 4.1 Diffusion Toolbox (http://www.fmrib.ox.ac.uk/fsl/) through a standard multistep procedure that incorporated both eddy current correction and brain extraction^1,2^. The “Eddy-correct” tool in FSL corrects for misregistration between successive images due to eddy currents or patient movement, through registration of each diffusion weighted image to a non-diffusion-weighted b0 image^1,2^. All processed DTI images were visually inspected for any misregistration and following brain extraction, the diffusion tensor was estimated at each image voxel using the “DTIFIT” tool within FSL.

**DCE-MRI acquisition**

DCE-MRI data were collected using a dual-injection, dual temporal resolution (DTR) technique, as described previously^3–5^*.* A macrocyclic gadolinium-based contrast agent (GBCA, gadoterate meglumine; Dotarem, Geurbet S.A.) was administered by power injector as an intravenous bolus at a rate of 3 ml/s, followed by a chaser of 20 ml of 0.9% saline administered at the same rate. For the first part of this DTR technique, a high temporal but low spatial resolution sequence with a low dose of GBCA (fixed volume of 3 ml) was performed (LDHT-DCE). The LDHT-DCE series used 3D GRE sequence with a flip angle of 16°, a field-of-view of 240 x 240 mm, image matrix of 96 x 96 x 22 voxels, and temporal resolution (Δ*t*) of 1.3 s (n = 300). Subsequently, a full GBCA dose (0.1 mmol/kg), high spatial but low temporal resolution (FDHS DCE) acquisition (voxel size of 1 x 1 x 2 mm; matrix size of 240 x 240 x 70, Δ*t* =10.1 s, n = 60) was performed to provide high spatial resolution data. Variable flip angle (VFA; *α* = 2°, 6°, 12° and 16°) acquisitions were performed prior to the LDHT and FDHS DCE series for native longitudinal relaxation rate (R1_N_) mapping. A post contrast high-resolution 3D T1-weighted gradient echo sequence of the whole brain was also obtained at the end of the DCE-MRI sequence to facilitate tumour delineation.

**DCE-MRI data analysis**

To permit high-spatial assessment of changes in tumour microvascular parameters following SRS treatment, a previously validated DCE-MRI analysis technique, termed LEGATOS (**LE**vel and rescale the **Ga**dolinium contrast concentrations curves of high-temporal **TO** high-**S**patial DCE-MRI) was adopted^4,5^. In traditional Cartesian MRI, it is difficult to simultaneously achieve both high spatial (HS) and high temporal (HT) resolution and a compromise must often be made in protocol design. In particular low temporal resolution during the early ‘arterial’ phase of the acquisition can significantly compromise the accuracy of parameter estimates due to undersampling of the bolus peak and temporal jitter uncertainty (uncertainty in the relative alignments of the vascular input function and tissue uptake curves)^5,6^.

The LEGATOS (**LE**vel and rescale the **Ga**dolinium contrast concentration curves of high-temporal **TO** high-**S**patial DCE-MRI) technique addresses this limitation of traditional Cartesian methods through two key steps. In key step 1, errors due to temporal jitter uncertainty are reduced through construction of a merged DTR 4D GBCA concentration volume containing a high temporal (HT) resolution ‘arterial’ phase followed by a later low temporal but high spatial (HS) resolution ‘parenchymal’ phase ^4–6^. In key step II the high temporal but low spatial resolution arterial phase of each pixel concentration curve is then re-scaled using the LEGATOS method and a derived pixelwise calibration ratio, to increase the spatial resolution of derived kinetic parameter maps. Further details on this analysis technique are provided in the included reference^5^.

**Kinetic analysis**

Following initial HT time course reconstruction (the LEGATOS method), the tissue GBCA concentration-time curves of the 4D HTHS-merged concentration volume were the fitted to the extended Tofts model. Derivation of DCE-MRI derived kinetic parameters through modelling requires identification of a suitable vascular input function (VIF) and a good approximation can be achieved by measuring the input function in the superior sagittal sinus (SSS)^7,8^. This vascular input function (VIF) measurement method has been previously described and it uses a semi‐automatic extraction method, to identify voxels within the SSS that display maximum enhancement during the first pass of the GBCA bolus^5,9,10^. For the LEGATOS reconstruction, a combined vascular input function is utilised. This VIF is constructed through concatenation of the early ‘arterial’ phase of the GBCA concentration-time curve, C_p_ (*t*), from the LDHT series with the later parenchymal phase of the dose calibrated GBCA concentration–time curve from the FDHS-DCE series^4,5^. Prior to concatenation, the amplitude of the C_p_ (*t*) from the FDHS series is scaled down to match the LDHT derived C_p_ (*t*) using the dose calibration ratio. An example concatenated VIF used for the DCE-MRI analysis is shown in *supplementary Figure S1* alongside an example fit to a tumour voxel using the extended Tofts model (ETM).

**^23^Na- MRI protocol**

^23^Na-MRI acquisition for TSC estimation was performed using a separate dual tuned ^1^H/^23^Na birdcage headcoil (RAPID Biomedical, Rimpar, Germany). The fast decaying transverse component (T2_F_ ≈ 0.5-5ms) of the sodium signal in vivo means that ultra-short echo time (UTE) sequences with non-Cartesian k-space sampling are typically used^11,12^. For this study a UTE spiral acquisition was performed with the following parameters: TE=0.967ms; TR=100ms, no. of averages, NOA=7; nominal isotropic voxel size= 4 mm; nominal field-of-view (FOV) of 240 × 240 mm^2^. Saturation of the longitudinal component of the sodium signal was mitigated by using a long TR (100ms) value compared to the T1 of tissue sodium (T1~35–40ms)^13^.

To allow for quantification of TSC in both tumour and normal appearing brain, two sodium calibration phantoms made from a stock of 60 mM and 120 mM sodium chloride (NaCl) in water with 4% agarose were used. Each of the phantoms were cylindrical tubes of 30 ml capacity (physical dimensions 2.45 × 8.9 cm; Dutscher, France), filled with the stock solution. These were placed beside the ear defenders/headphones worn by the subjects to include them in the imaging FOV^12,14^. T1 measurements in each phantom (measured through inversion recovery experiments) were in good agreement with reported literature values. Regular quality assurance scans using the phantoms and commercial 0.9% saline preparations (Baxter Healthcare Inc, Deerfield, IL, USA) as a calibrant demonstrated that the concentrations within both phantoms were stable over the course of the study, with mean concentrations of 56 ± 3mM and 123 ± 4mM respectively.

**^23^Na- MRI analysis**

The spiral ^23^Na-MRI images were reconstructed on the scanner and the TSC maps were subsequently calculated offline using MATLAB 2015a (The MathWorks, Inc., Natick, MA, USA)^15^. Noise and power correction of sodium images was undertaken using the method described by Miller et al^16,17^. Prior to calculation of the TSC map the measured intensities from each phantom placed in the FOV were divided by a correction factor ( f =0.96) to take into account the fact that T1 relaxation in the phantom is incomplete due to the relatively short TR of the ^23^Na-MRI acquisition^15,18,19^

**References:**

1. Smith, S. M. Fast robust automated brain extraction. *Hum. Brain Mapp.* **17**, 143–155 (2002).

2. Soares, J. M., Marques, P., Alves, V. & Sousa, N. A hitchhiker’s guide to diffusion tensor imaging. *Front. Neurosci.* **7**, 31 (2013).

3. Li, K.-L. *et al.* An improved coverage and spatial resolution-using dual injection dynamic contrast-enhanced (ICE-DICE) MRI: A novel dynamic contrast-enhanced technique for cerebral tumors. *Magn. Reson. Med.* **68**, 452–462 (2012).

4. Li, K.-L., Lewis, D., Jackson, A., Zhao, S. & Zhu, X. Improving quantification accuracy in whole brain high spatial resolution 3D kinetic mapping: Development of a novel dual temporal resolution DCE-MRI technique. In Proceedings of the 27th Annual Meeting of ISMRM, Montreal, Canada, 2019. p. 4409. (2019).

5. Li, K.-L. *et al.* The LEGATOS technique: A new tissue-validated dynamic contrast-enhanced MRI method for whole-brain, high-spatial resolution parametric mapping. *Magn. Reson. Med.* (2021) doi:10.1002/mrm.28842.

6. Henderson, E., Rutt, B. K. & Lee, T. Y. Temporal sampling requirements for the tracer kinetics modeling of breast disease. *Magn. Reson. Imaging* **16**, 1057–1073 (1998).

7. Koenig, M. *et al.* Perfusion CT of the brain: Diagnostic approach for early detection of ischemic stroke. *Radiology* **209**, 85–93 (1998).

8. Keil, V. C. *et al.* Effects of arterial input function selection on kinetic parameters in brain dynamic contrast-enhanced MRI. *Magn. Reson. Imaging* **40**, 83–90 (2017).

9. Li, K. L., Lewis, D., Jackson, A., Zhao, S. & Zhu, X. Low-dose T1W DCE-MRI for early time points perfusion measurement in patients with intracranial tumors: A pilot study applying the microsphere model to measure absolute cerebral blood flow. *J. Magn. Reson. Imaging* **48**, 543–557 (2018).

10. Li, K. L., Zhu, X., Zhao, S. & Jackson, A. Blood–brain barrier permeability of normal-appearing white matter in patients with vestibular schwannoma: A new hybrid approach for analysis of T1-W DCE-MRI. *J. Magn. Reson. Imaging* **46**, 79–93 (2017).

11. Ouwerkerk, R., Bleich, K. B., Gillen, J. S., Pomper, M. G. & Bottomley, P. a. Tissue sodium concentration in human brain tumors as measured with 23Na MR imaging. *Radiology* **227**, 529–537 (2003).

12. Riemer, F. *et al.* Sodium (23Na) ultra-short echo time imaging in the human brain using a 3D-Cones trajectory. *Magn. Reson. Mater. Physics, Biol. Med.* **27**, 35–46 (2014).

13. Thulborn, K. *et al.* Quantitative sodium MRI of the human brain at 9.4 T provides assessment of tissue sodium concentration and cell volume fraction during normal aging. *NMR Biomed.* **29**, 137–143 (2016).

14. Riemer, F. *et al.* Measuring tissue sodium concentration: Cross-vendor repeatability and reproducibility of 23Na-MRI across two sites. *J. Magn. Reson. Imaging* **50**, 1278–1284 (2019).

15. Christensen, J. D., Barrère, B. J., Boada, F. E., Vevea, J. M. & Thulborn, K. R. Quantitative tissue sodium concentration mapping of normal rat brain. *Magn. Reson. Med.* **36**, 83–89 (1996).

16. Newbould, R. D. *et al.* Reproducibility of sodium MRI measures of articular cartilage of the knee in osteoarthritis. *Osteoarthr. Cartil.* **20**, 29–35 (2012).

17. Riemer, F., Solanky, B. S., Wheeler-Kingshott, C. A. M. & Golay, X. Bi-exponential 23Na T2* component analysis in the human brain. *NMR Biomed.* **31**, (2018).

18. Inglese, M. *et al.* Brain tissue sodium concentration in multiple sclerosis: a sodium imaging study at 3 tesla. *A J. Neurol.* doi:10.1093/brain/awp334.

19. Paling, D. *et al.* Sodium accumulation is associated with disability and a progressive course in multiple sclerosis. *Brain* **136**, (2013).

# Supplementary figures

**
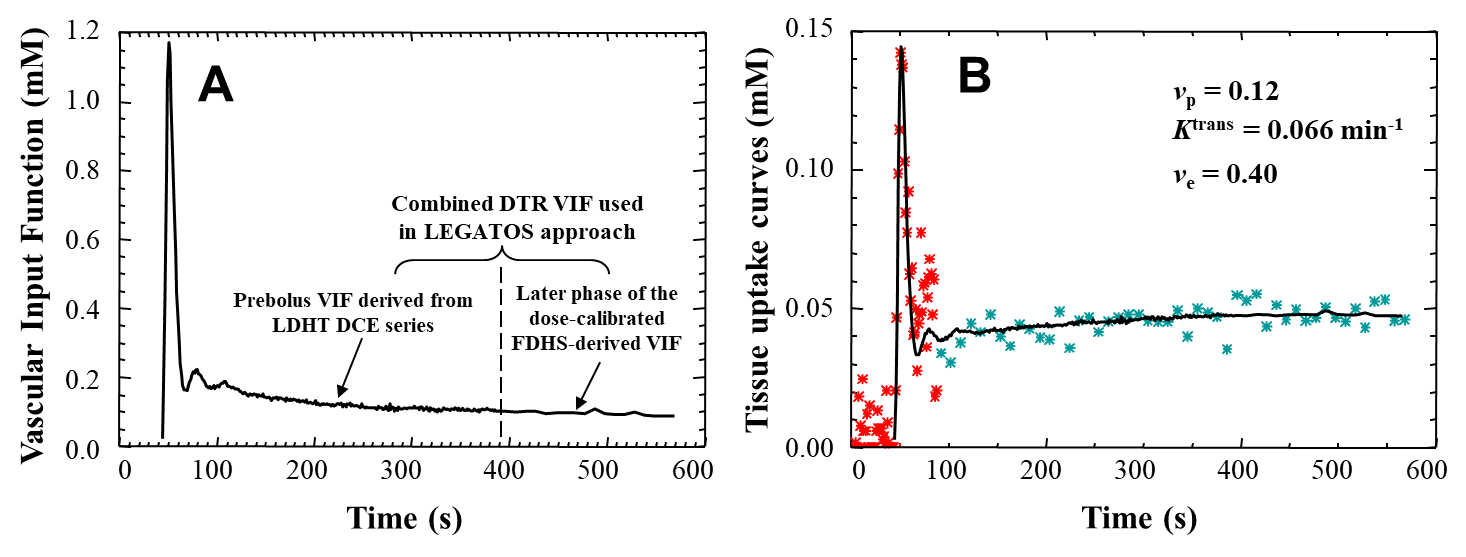
**

**Supplementary Fig.S1: Derived vascular input function (VIF) from the superior sagittal sinus (SSS) and example fit to a tumour voxel**

**A:** Combined vascular input function (VIF) used for the LEGATOS analysis of dual injection, dual temporal resolution (DTR) DCE-MRI data. The combined VIF is derived from voxels within the SSS and constructed through concatenation of the early ‘arterial’ phase of the GBCA concentration-time curve ,C_p_ (*t*), from the LDHT-DCE series with the later ‘parenchymal’ phase of the dose calibrated concentration–time curve from the FDHS-DCE series^4,5^. Prior to concatenation, the amplitude of the C_p_ (*t*) from the FDHS-DCE series is scaled down to match the C_p_ (*t*) from the LDHT-DCE series using the dose calibration ratio. The VIF shown was extracted from voxels within the SSS of a 79yr old patient with a left-sided, sporadic VS who underwent dual injection (3 ml and 13.8 ml of contrast agent, respectively) DTR DCE MRI at 3T. **B:** GBCA concentration – time curve from a representative tumour voxel. The LEGATOS reconstructed arterial phase of the low-dose HT_aligned_ GBCA concentration-time course obtained from the LDHT-DCE series (*red asterisk*) was concatenated with the dose-calibrated HS parenchymal tissue phase obtained from the FDHS-DCE series (*blue asterisk*). The theoretical fit obtained using the extended Tofts model (ETM) for the voxel curve is shown (*solid line*) along with the derived parameter estimates.

**
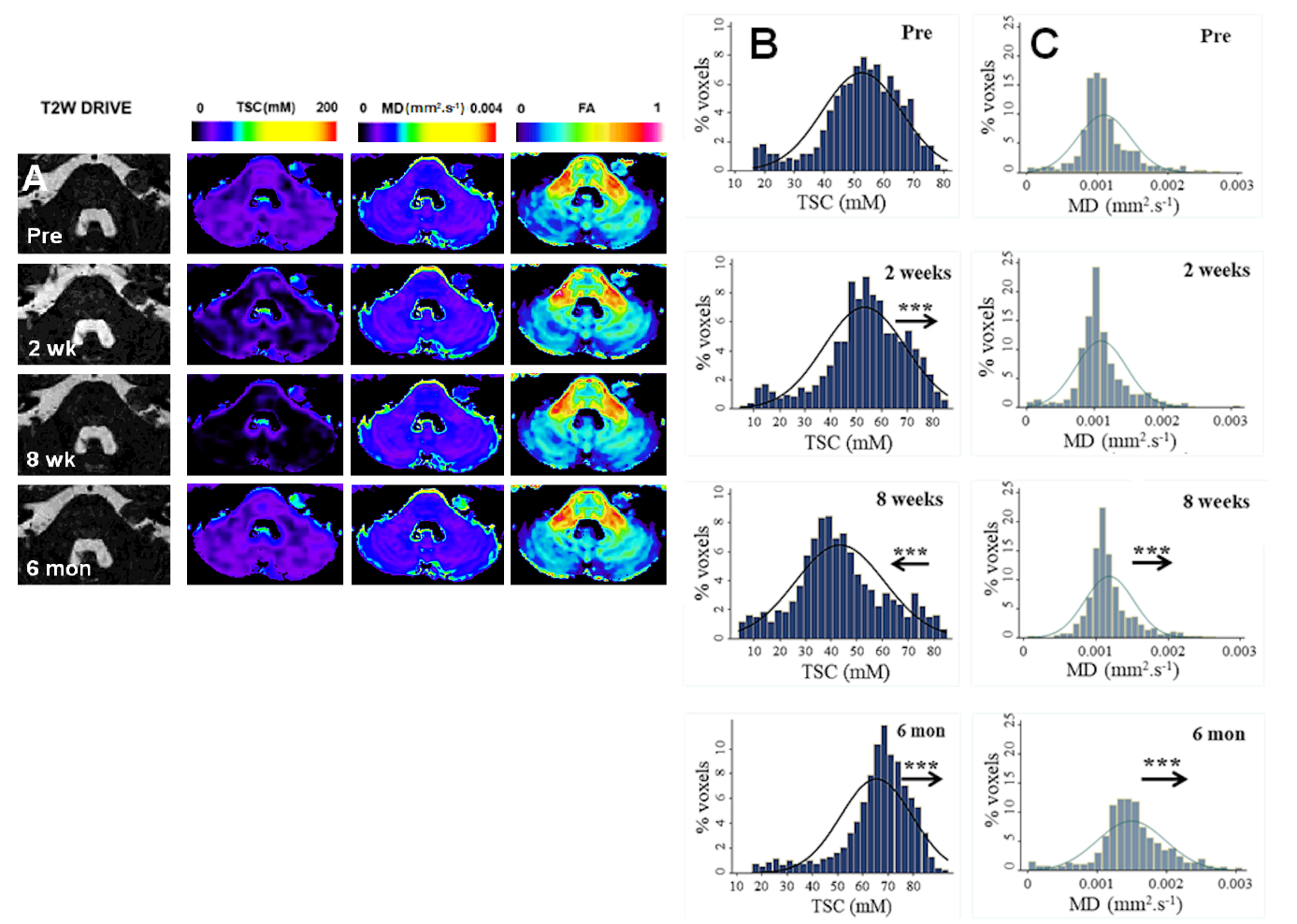
**

**Supplementary Fig.S2: Post-SRS changes in tumour total sodium concentration (TSC) and diffusion metrics in patient 5**

**A:** Representative TSC and diffusion metric maps from a 79 yr old patient with left-sided growing VS (patient 5). From left to right: T2W-DRIVE, T1W post-contrast, co-registered voxelwise maps of TSC, MD and FA. T2W-DRIVE acquisition demonstrates the VS within the left cerebellopontine angle and the absence of longitudinal macroscopic structural change at all timepoints. Voxelwise maps of TSC demonstrate the early increase in TSC at 2 weeks and subsequent decrease at 8 weeks post-SRS. At 6 months post-SRS there are demonstrable increases in TSC and MD and reductions in tumour FA. **B:** Histogram of voxelwise TSC values within the tumour. From top: pre-treatment, 2 weeks post-treatment, 8 weeks post-treatment and 6 months post-treatment. Note the early increase and subsequent decrease in TSC at 2 weeks and 8 weeks post-SRS respectively. **C:** Histogram of voxelwise MD values within the tumour. From top: pre-treatment, 2 weeks post-treatment, 8 weeks post-treatment and 6 months post-treatment. Note that significant voxelwise changes in MD are not present until 8 weeks post-SRS. *** p<0.001 for comparison with pre-treatment TSC and MD values. Repeated measures ANOVA with post hoc (Bonferroni) analysis of pairwise comparison. Arrows show movement in histogram relative to preceding imaging timepoint. FA=Fractional anisotropy; MD=Mean diffusivity; TSC=Total sodium concentration


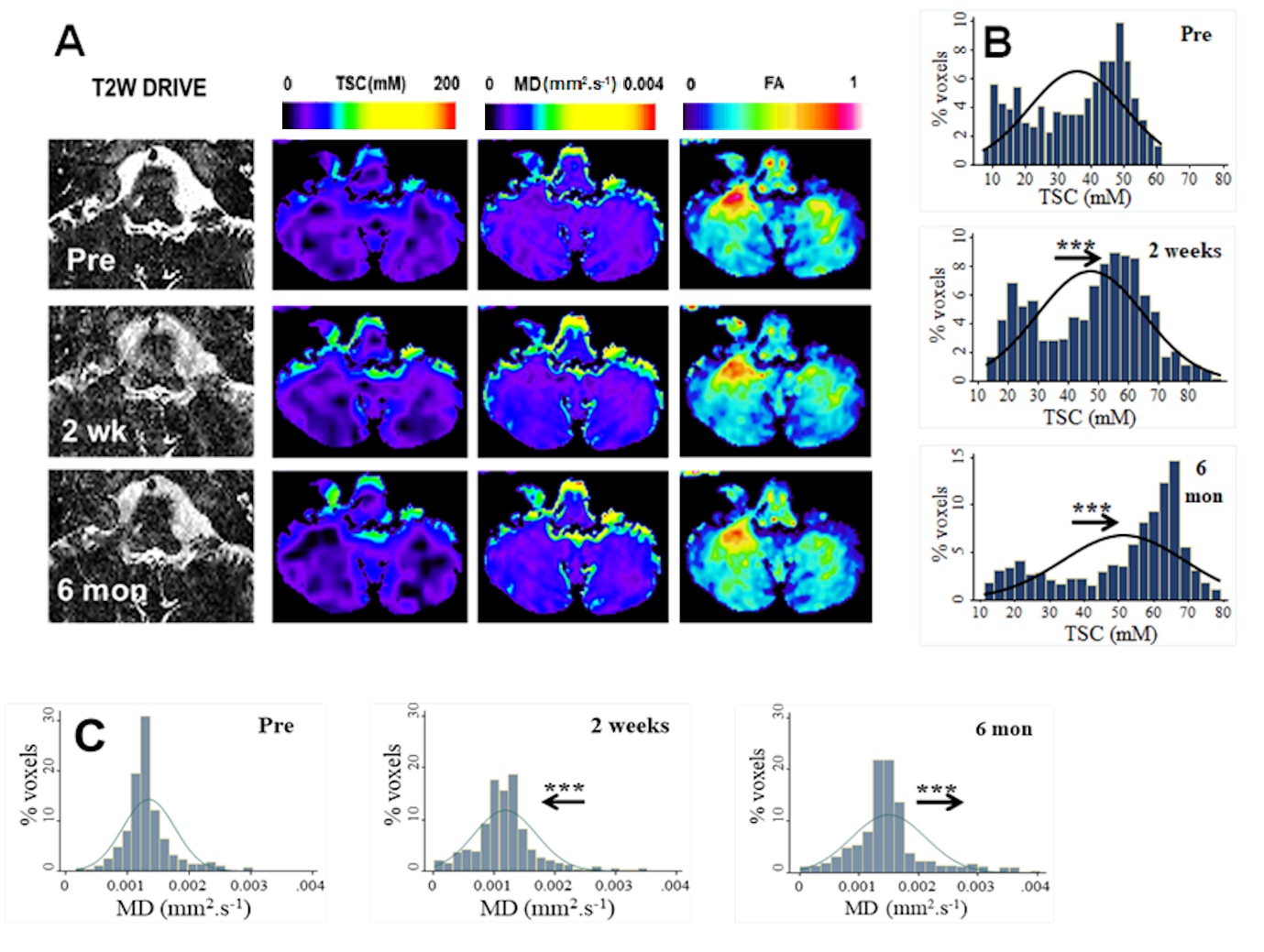


**Supplementary Fig.S3: Post-SRS changes in tumour total sodium concentration (TSC) and diffusion metrics in patient 3**

**A:** Representative TSC and diffusion metric maps from a 69 yr old patient with a right-sided growing VS (patient 3). From left to right: T2W-DRIVE, co-registered voxelwise maps of TSC, MD and FA. T2W-DRIVE acquisition demonstrates the VS within the left cerebellopontine angle and the absence of longitudinal macroscopic structural change within the tumour. Voxelwise maps of TSC demonstrate the early increase in TSC at 2 weeks and 6 months post-SRS. Note the later increase in MD and reduction in tumour FA at 6 months post-SRS. **B:** Histogram of voxelwise TSC values within the tumour. Pre-treatment (top), 2 weeks post-treatment (middle) and 6 months post-treatment (bottom). Note the upward shift in voxelwise TSC values at 2 weeks and 6 months post-treatment. **C:** Histogram of voxelwise MD values within the tumour. From left to right: Pre-treatment, 2 weeks post-treatment and 6 months post-treatment. Note the early decrease in voxelwise MD values at 2 weeks and subsequent increase at 6 months post-treatment. *** p<0.001 for comparison with pre-treatment TSC values. Repeated measures ANOVA with post hoc (Bonferroni) analysis of pairwise comparison. Arrows show movement in histogram relative to preceding imaging timepoint. MD=Mean diffusivity; FA=Fractional anisotropy; TSC=Total sodium concentration


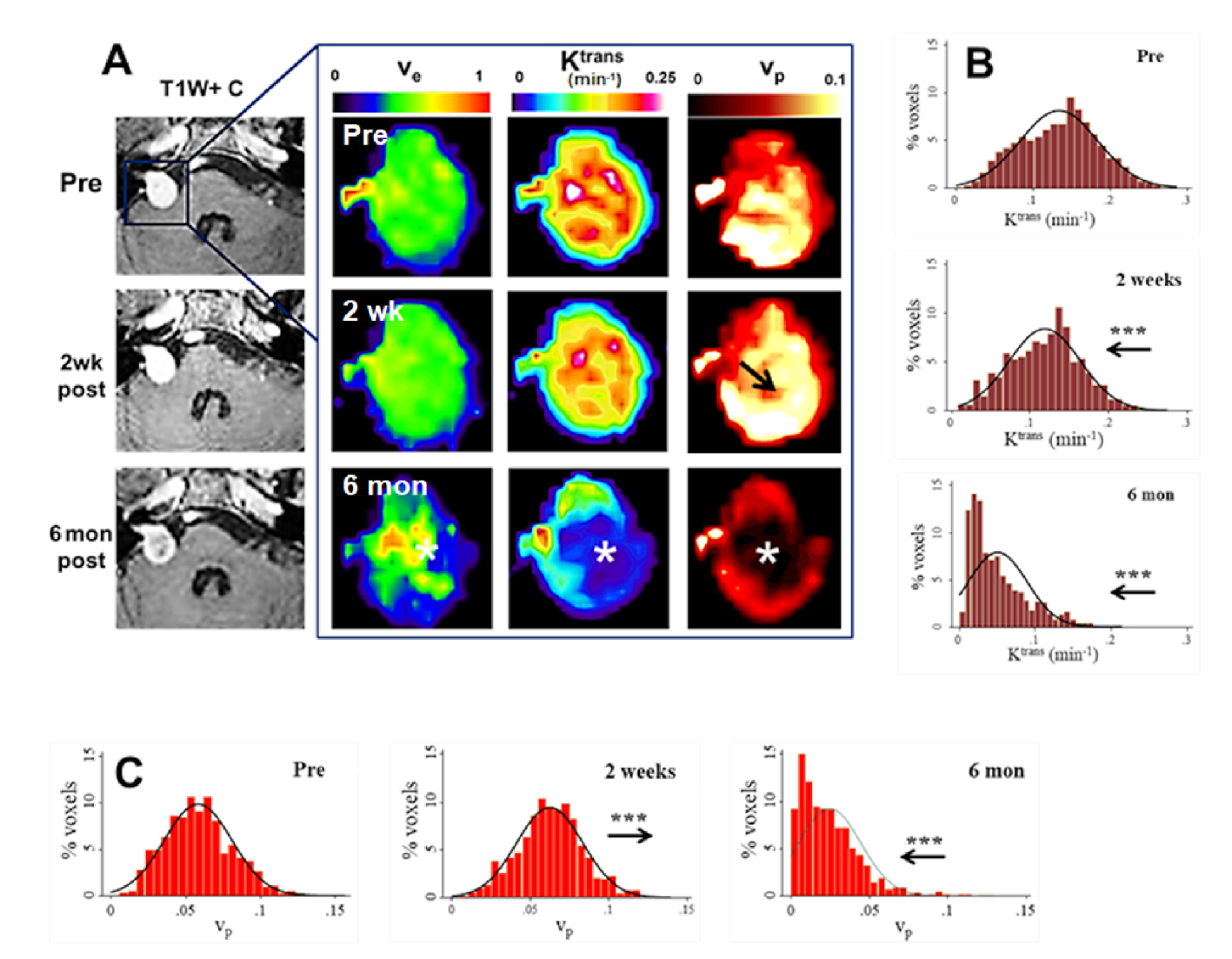


**Supplementary Fig.S4: Post-SRS changes in tumour microvascular parameters in patient 4**

**A:** Representative parameter maps from a 73 yr old patient with a right-sided growing VS undergoing SRS (patient 4). From left to right: T1W post-contrast, co-registered voxelwise maps of v_e_, K^trans^, and v_p_ derived from DCE-MRI. Note the observable increases in v_p_ at 2 weeks (*arrow*) and the subsequent marked decrease in v_e_, K^trans^ and v_p_ within the CPA portion of the tumour at 6 months post-SRS (*). **B:** Histogram of voxelwise K^trans^ values within the tumour pre-treatment (top), 2 weeks post-treatment (middle) and 6 months post-treatment (bottom). Note the decrease in voxelwise K^trans^ values at 2 weeks and 6 months post-treatment. **C:** Histogram of voxelwise v_p_ values within the tumour. From left to right: pre-treatment, 2 weeks post-treatment and 6 months post-treatment. Note the increase in voxelwise v_p_ values at 2 weeks and marked decrease at 6 months post-treatment. *** p<0.001 for comparison with pre-treatment TSC values. Repeated measures ANOVA with post hoc (Bonferroni) analysis of pairwise comparison. Arrows show movement in histogram relative to preceding imaging timepoint.

**
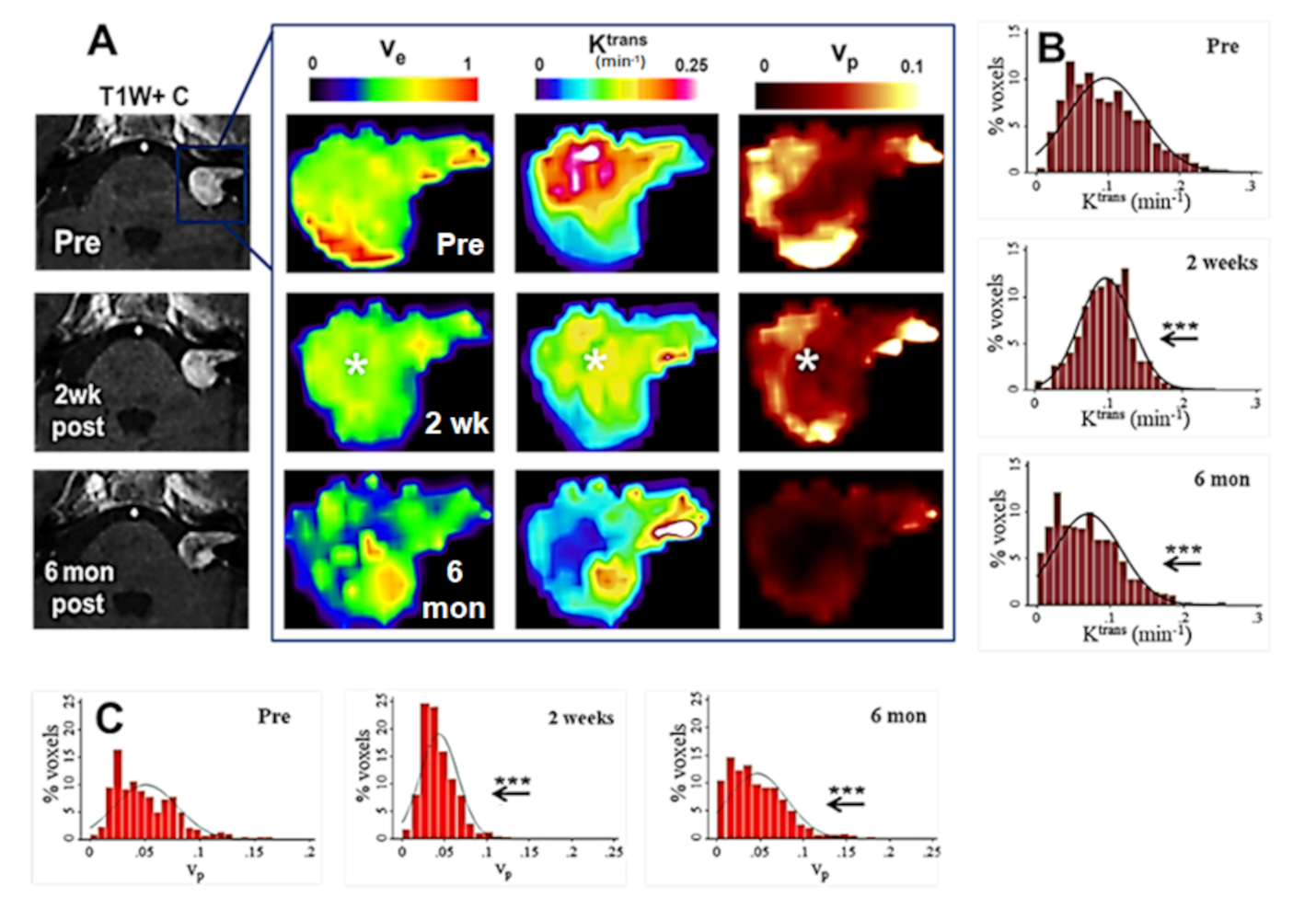
**

**Supplementary Fig.S5: Post-SRS changes in tumour microvascular parameters in patient 5**

**A:** Representative parameter maps from a 79 yr old patient with a left-sided growing VS undergoing SRS (patient 5). From left to right: T1W post-contrast, co-registered voxelwise maps of v_e_, K^trans^, and v_p_ derived from DCE-MRI. Note the decreases in v_e_, K^trans^ and v_p_ at 2 weeks (*) and 6 months post-SRS. **B:** Histogram of voxelwise K^trans^ values within the tumour pre-treatment (top), 2 weeks post-treatment (middle) and 6 months post-treatment (bottom). Note the downward shift in voxelwise K^trans^ values at 2 weeks and 6 months post-treatment. **C:** Histogram of voxelwise v_p_ values within the tumour. From left to right: pre-treatment, 2 weeks post-treatment and 6 months post-treatment. Note the downward shift in voxelwise v_p_ values at 2 weeks and 6 months post-treatment. *** p<0.001 for comparison with pre-treatment TSC values. Repeated measures ANOVA with post hoc (Bonferroni) analysis of pairwise comparison. Arrows show movement in histogram relative to preceding imaging timepoint.
