# Supplementary material for: Detection of early changes in the post-radiosurgery vestibular schwannoma microenvironment using multinuclear MRI
Source: Sci Rep. 2021 Aug 3;11:15712. doi: 10.1038/s41598-021-95022-6 (PMC8333359; doi:10.1038/s41598-021-95022-6)
Supplement: Supplementary file 2 — Supplementary Tables. [file 41598_2021_95022_MOESM2_ESM.docx]

**Detection of early changes in the post-radiosurgery vestibular schwannoma microenvironment using multinuclear MRI**

**Daniel Lewis MRCS^1,2,3*^, Damien J McHugh PhD^4^, Ka-loh Li PhD^3^, Xiaoping Zhu PhD^3^, Catherine Mcbain MD^2,5^,** [**Simon K. Lloyd**](https://thejns.org/search?f_0=author&q_0=Simon+K.+Lloyd)**FRCS(ORL-HNS)^6^, Alan Jackson PhD^3^, Omar N Pathmanaban PhD^1,2,7^, Andrew T King FRCS (SN)^1,2,8^, David J Coope PhD^1,2^**

# Supplementary tables

**Supplementary Table S1: Total sodium concentration (TSC) in atlas defined normal appearing brain regions**

P value for differences in TSC calculated using repeated measures mixed-effects model. Post hoc analysis of pairwise comparisons between different timepoints was performed using the Bonferroni method.

| **Patient** | **GM [TSC, all]** | | | | **WM [TSC, all]** | | | | **CSF [TSC, all]** | | | |
| --- | --- | --- | --- | --- | --- | --- | --- | --- | --- | --- | --- | --- |
|  | **Pre** | **2**  **weeks** | **8**  **weeks** | **6 months** | **Pre** | **2**  **weeks** | **8**  **weeks** | **6 months** | **Pre** | **2**  **weeks** | **8**  **weeks** | **6**  **months** |
| 1 | 39.3 | 41.9 | 35.5 |  | 32.3 | 36.1 | 29.3 |  | 66.8 | 68.4 | 63.3 |  |
| 2 |  | 47.0 | 41.4 | 41.5 |  | 44.4 | 37.8 | 38.2 |  | 64.5 | 62.6 | 59.6 |
| 3 | 36.0 | 37.2 |  | 32.9 | 35.5 | 37.5 |  | 35.1 | 67.1 | 75.8 |  | 69.8 |
| 4 | 43.5 | 50.5 | 49.1 | 46.1 | 37.8 | 45.1 | 44.9 | 41.4 | 73.2 | 80.7 | 75.5 | 76.7 |
| 5 | 38.6 | 33.1 | 28.4 | 41.5 | 35.8 | 29.8 | 25.1 | 39.6 | 72.3 | 75.1 | 72.2 | 76.6 |
| **Mean**  **(±SD)** | 39.4  (3.11) | 42.0  (7.03) | 38.6  (8.81) | 40.5  (5.50) | 35.4  (2.27) | 38.6  (6.36) | 34.3  (8.84) | 38.6  (2.68) | 69.9  (3.39) | 72.9  (6.41) | 68.4  (6.43) | 70.7  (8.05) |
| **Mean difference from baseline (95% CI)** | | 2.26  (-6, 10) | -3.57  (-66, 59) | 0.75  (-4, 5) |  | 3.02  (-3, 10) | -1.90  (-9, 5) | 2.57  (-0.7,5) |  | 4.24  (-12, 3) | -0.21  (-5, 5) | 0.75  (-13, 15) |
| **P value** | | 0.42 | 0.51 | 0.67 |  | 0.28 | 0.59 | 0.10 |  | 0.29 | 0.99 | 0.99 |

**Supplementary Table S2: Total sodium concentration (TSC) in manual ROI defined normal appearing brain regions**

P value for differences in TSC calculated using repeated measures mixed-effects model. Post hoc analysis of pairwise comparisons between different timepoints was performed using the Bonferroni method.

| **Patient** | **WM [centrum semiovale]** | | | | **CSF [lateral ventricle]** | | | |
| --- | --- | --- | --- | --- | --- | --- | --- | --- |
|  | **Pre** | **2 weeks** | **8 weeks** | **6 months** | **Pre** | **2 weeks** | **8 weeks** | **6 months** |
| 1 | 30.4 | 35.4 | 28.3 |  | 110.9 | 112.7 | 107.3 |  |
| 2 |  | 49.2 | 40.8 | 38.5 |  | 95.3 | 94.4 | 84.7 |
| 3 | 35.1 | 43.0 |  | 36.6 | 116.1 | 132.2 |  | 127.0 |
| 4 | 39.2 | 45.8 | 46.2 | 41.7 | 119.3 | 133.6 | 122.1 | 130.8 |
| 5 | 38.5 | 39.5 | 31.1 | 47.1 | 109.1 | 119.6 | 116.6 | 114.9 |
| **All** | 35.8  (4.03) | 42.6  (5.34) | 36.6  (8.36) | 41.0  (4.58) | 113  (4.71) | 119  (15.7) | 110  (12.1) | 114  (20.8) |
| **Mean difference from baseline**  **(95% CI)** | | **6.4**  **(1, 12)** | 0.09  (-15.3) | 3.09  (-6,12) |  | 7.9  (-0.7, 16) | 1.5  (-4,7) | 2.43  (-8,13) |
| **P value** | | **0.03** | 0.98 | 0.32 |  | 0.07 | 0.51 | 0.63 |

**Supplementary Table S3: Repeatability of normal brain total sodium concentration (TSC, mM) estimates from atlas defined and manual ROI defined normal appearing brain regions**

Individual patient level coefficient of variation (CoV) values reported alongside mean (+/- S.D) and global CoV.

^a^Average measures ICC estimates are reported based on an absolute-agreement, 2-way mixed-effects model. Data from three timepoints used in calculation for each patient.

| **Patient** | **GM [all]** | **WM [all]** | **CSF [all]** | **CSF [lat vent]** | **WM [CS]** |
| --- | --- | --- | --- | --- | --- |
| **1** | 8.26 | 10.4 | 3.97 | 2.48 | 11.7 |
| **2** | 7.34 | 9.25 | 4.00 | 6.40 | 13.1 |
| **3** | 6.29 | 3.66 | 6.26 | 6.57 | 11.0 |
| **4** | 6.60 | 8.18 | 4.10 | 5.39 | 7.80 |
| **5** | 16.5 | 19.7 | 2.91 | 3.83 | 16.8 |
| **Mean CoV ±SD** | 9.00  (4.26) | 10.2  (5.87) | 4.25  (1.22) | 4.93  (1.75) | 12.1  (3.27) |
| **Global CoV** | 9.77 | 11.5 | 4.39 | 5.18 | 12.4 |
| **ICC^a^** | 0.81 | 0.65 | 0.91 | 0.94 | 0.71 |

**Supplementary Table S4: Temporal changes in whole tumour ROI total sodium concentration (TSC), relative TSC ratio and diffusion metrics (MD, FA)**

P value calculated using repeated measures mixed-effects model. Post hoc analysis of pairwise comparisons between different timepoints was performed using the Bonferroni method.

| **Patient** | **Mean tumour TSC (mM)** | | | | **Mean tumour-to-CSF TSC ratio** | | | | **Mean diffusivity**  **(MD, mm^2^.s^-1^ x10^-6^)** | | | | | **Mean fractional**  **anisotropy (FA)** | | | |
| --- | --- | --- | --- | --- | --- | --- | --- | --- | --- | --- | --- | --- | --- | --- | --- | --- | --- |
|  | **Pre** | **2 weeks** | **8 weeks** | **6**  **months** | **Pre** | **2 weeks** | **8 weeks** | **6 months** | **Pre** | **2**  **weeks** | **8 weeks** | **6 months** | **Pre** | | **2**  **weeks** | **8**  **weeks** | **6**  **months** |
| 1 | 57.6 | 60.9 | 58.8 |  | 0.86 | 0.89 | 0.93 |  | 1320.3 | 1396.91 | 1373.2 |  | 0.25 | | 0.20 | 0.21 |  |
| 2 |  | 65.1 | 64.3 | 60.3 |  | 1.01 | 1.03 | 1.01 |  | 1218.6 | 1193.3 | 1354.9 |  | | 0.35 | 0.34 | 0.24 |
| 3 | 36.0 | 47.9 |  | 51.3 | 0.54 | 0.63 |  | 0.74 | 1333.3 | 1134.0 |  | 1464.9 | 0.24 | | 0.33 |  | 0.21 |
| 4 | 55.2 | 68.0 | 64.9 | 71.1 | 0.75 | 0.84 | 0.86 | 0.93 | 1246.5 | 1287.4 | 1242.6 | 1485.2 | 0.24 | | 0.24 | 0.21 | 0.21 |
| 5 | 51.7 | 52.4 | 42.6 | 64.6 | 0.72 | 0.70 | 0.59 | 0.84 | 1075.6 | 1070.7 | 1175.0 | 1460.2 | 0.28 | | 0.28 | 0.25 | 0.19 |
| **Mean ±SD** | 50.1  (26.6) | 58.9  (8.88) | 57.7  (3.36) | 61.8  (9.91) | 0.72  (0.14) | 0.81  (0.15) | 0.85  (0.19) | 0.88  (0.12) | 1221.0 | 1200.0 | 1215.6 | 1441.3 | 0.25 | | 0.28 | 0.25 | 0.21 |
| **Mean difference from baseline (95% CI)** | | 7.91  (-1.52,  17.3) | 1.96  (-11.9,  15.8) | **11.7**  **(1.77,**  **21.6)** |  | 0.06  (-0.02, 0.13) | 0.006  (-0.25,  0.15) | **0.14**  **(0.03,**  **0.24)** |  | -8.15  (-124, 109) | 8.84  (-90, 107) | **241**  **(135, 347)** |  | | 0.02  (-0.04,0.08) | -0.005  (-0.06, 0.05) | **-0.05**  **(-0.08,-0.03)** |
| **P value** | | 0.12 | 0.99 | **0.02** |  | 0.16 | 0.99 | **0.002** |  | 0.88 | 0.86 | **<0.001** |  |  | 0.41 | 0.80 | **0.003** |

**Supplementary Table S5: Temporal changes in DCE-MRI derived microvascular parameters of reference muscle ROI**

P value calculated using repeated measures mixed-effects model. Post hoc analysis of pairwise comparisons between different timepoints was performed using the Bonferroni method. Intra-subject variability of v_e_, K^trans^ and v_p_ values across each imaging timepoint shown using the mean (+/- S.D) and global coefficient of variation (CoV).

| **Patient** | **Muscle ROI v_e_**  **(no units)** | | | **Muscle ROI K^trans^,**  **min^-1^** | | | **Muscle ROI v_p_**  **(no units)** | | |
| --- | --- | --- | --- | --- | --- | --- | --- | --- | --- |
|  | **Pre** | **2 weeks** | **6 months** | **Pre** | **2 weeks** | **6 months** | **Pre** | **2 weeks** | **6 months** |
| 1 | 0.29 | 0.26 |  | 0.08 | 0.09 |  | 0.02 | 0.02 |  |
| 2 |  | 0.31 | 0.36 |  | 0.08 | 0.13 |  | 0.02 | 0.04 |
| 3 | 0.39 | 0.34 | 0.38 | 0.06 | 0.07 | 0.04 | 0.01 | 0.02 | 0.02 |
| 4 | 0.32 | 0.23 | 0.20 | 0.05 | 0.04 | 0.08 | 0.02 | 0.02 | 0.01 |
| 5 | 0.34 | 0.35 | 0.29 | 0.11 | 0.10 | 0.15 | 0.02 | 0.02 | 0.03 |
| **Mean (±SD)** | 0.33  (0.04) | 0.30  (0.05) | 0.31  (0.08) | 0.08  (0.03) | 0.08  (0.02) | 0.10  (0.05) | 0.02  (0.00) | 0.02  (0.00) | 0.02  (0.01) |
| **Mean difference from baseline**  **(95% CI)** |  | -0.04  (-0.09, 0.02) | -0.05  (-0.16, 0.06) |  | -0.002  (-0.03, 0.03) | 0.03  (-0.03, 0.08) |  | 0.001  (-0.01, 0.01) | 0.007  (-0.02, 0.03) |
| **P value** |  | 0.21 | 0.49 |  | 0.99 | 0.55 |  | 0.99 | 0.99 |
| **Mean CoV (%) ±SD** | 11.8  (7.35) | | | 23.2  (12.0) | | | 27.2  (21.7) | | |
| **Global CoV** | 13.6 | | | 25.6 | | | 33.4 | | |

**Supplementary Table S6: Temporal changes in whole tumour ROI DCE-MRI derived microvascular parameters**

P value calculated using repeated measures mixed-effects model. Post hoc analysis of pairwise comparisons between different timepoints was performed using the Bonferroni method.

| **Patient** | **Mean tumour v_e_**  **(no units)** | | | **Mean tumour K^trans^,**  **min^-1^** | | | **Mean tumour v_p_**  **(no units)** | | |
| --- | --- | --- | --- | --- | --- | --- | --- | --- | --- |
|  | **Pre** | **2 weeks** | **6 months** | **Pre** | **2 weeks** | **6 months** | **Pre** | **2 weeks** | **6 months** |
| 1 | 0.58 | 0.68 |  | 0.13 | 0.17 |  | 0.04 | 0.06 |  |
| 2 |  | 0.38 | 0.36 |  | 0.13 | 0.07 |  | 0.08 | 0.06 |
| 3 | 0.41 | 0.42 | 0.22 | 0.16 | 0.20 | 0.05 | 0.07 | 0.10 | 0.04 |
| 4 | 0.36 | 0.37 | 0.35 | 0.13 | 0.12 | 0.05 | 0.06 | 0.06 | 0.02 |
| 5 | 0.51 | 0.45 | 0.38 | 0.10 | 0.10 | 0.07 | 0.05 | 0.04 | 0.04 |
| **Mean (±SD)** | 0.47  (0.10) | 0.46  (0.13) | 0.33  (0.07) | 0.13  (0.03) | 0.14  (0.04) | 0.06  (0.01) | 0.05  (0.01) | 0.07  (0.02) | 0.04  (0.02) |
| **Mean difference from baseline**  **(95% CI)** | | 0.002  (-0.16, 0.16) | -0.10  (-0.25, 0.06) |  | 0.01  (-0.08,0.10) | **-0.07**  **(-0.11,-0.04)** |  | 0.01  (-0.02, 0.04) | -0.02  (-0.05, 0.01) |
| **P value** | | 0.99 | 0.18 |  | 0.61 | **<0.001** |  | 0.91 | 0.31 |

Supplementary Table S7: Voxelwise correlation analysis between tumour total sodium concentration (TSC), diffusion metrics (MD, FA) and DCE-MRI derived microvascular parameters (v_e_, K^trans^ and v_p_)

Voxelwise correlation coefficient derived using either all patient datasets (*) or two patient datasets (**) with complete follow-up imaging (patient 4 and 5). Spearman’s rho shown.

| **Patient** | **TSC: MD correlation** | | | | **TSC: FA correlation** | | | | **TSC : v_e_** | | | **TSC: K^trans^** | | | **TSC: v_p_** | | |
| --- | --- | --- | --- | --- | --- | --- | --- | --- | --- | --- | --- | --- | --- | --- | --- | --- | --- |
|  | **Pre** | **2 weeks** | **8 weeks** | **6 mon** | **Pre** | **2 weeks** | **8 weeks** | **6 mon** | **Pre** | **2 weeks** | **6 mon** | **Pre** | **2**  **weeks** | **6 mon** | **Pre** | **2**  **weeks** | **6 mon** |
| 1 | 0.54  P<0.001 | 0.50  P<0.001 | 0.53  P<0.001 |  | -0.58  P<0.001 | -0.35  P<0.001 | -0.56  P<0.001 |  | 0.02  P=0.68 | -0.21  P0.002 |  | -0.06  P=0.18 | -0.24  P<0.01 |  | -0.56  P<0.001 | -0.32  P<0.001 |  |
| 2 |  | 0.02  P=0.67 | 0.05  P=0.20 | 0.01  P=0.89 |  | 0.01  P=0.73 | 0.07  P=0.07 | -0.32  P<0.001 |  | 0.11  P=0.003 | -0.003  P=0.93 |  | -0.31  P<0.001 | -0.23  P<0.001 |  | -0.07  P=0.07 | -0.17  P<0.001 |
| 3 | 0.51  P<0.001 | 0.65  P<0.001 |  | 0.73  P<0.001 | -0.67  P<0.001 | -0.62  P<0.001 |  | -0.53  P<0.001 | -0.63  P<0.001 | -0.58  P<0.001 | -0.53  P<0.001 | 0.21  P<0.001 | -0.51  P<0.001 | -0.80  P<0.001 | 0.09  P=0.03 | -0.24  P<0.001 | -0.73  P<0.001 |
| 4 | 0.55  P<0.001 | 0.62  P<0.001 | 0.65  P<0.001 | 0.56  P<0.001 | -0.34  P<0.001 | -0.38  P<0.001 | -0.51  P<0.001 | -0.50  P<0.001 | -0.08  P=0.02 | -0.25  P<0.001 | -0.14  P<0.001 | 0.12  P<0.001 | 0.15  P<0.001 | -0.21  P<0.001 | -0.15  P<0.001 | -0.04  P=0.17 | -0.20  P<0.001 |
| 5 | 0.46  P<0.001 | 0.50  P<0.001 | 0.39  P=0.05 | 0.48  P<0.001 | -0.29  P<0.001 | -0.39  P<0.001 | -0.39  P<0.001 | -0.31  P<0.001 | -0.30  P<0.001 | -0.06  P=0.06 | -0.11  P=0.001 | -0.007  P=0.84 | -0.07  P=0.05 | -0.31  P<0.001 | -0.09  P=0.007 | -0.13  P<0.001 | -0.28  P<0.001 |
| **All**  ***** | **0.41**  **P<0.001** | **0.51**  **P<0.001** | **0.44**  **P<0.001** | **0.45**  **P<0.001** | **-0.37**  **P<0.001** | **-0.34**  **P<0.001** | **-0.28**  **P<0.001** | **-0.41**  **P<0.001** | **-0.06**  **P<0.001** | **-0.14**  **P<0.001** | **-0.002**  **P=0.92** | **-0.04**  **P=0.03** | **-0.11**  **P<0.001** | **-0.24**  **P<0.001** | **-0.27**  **P<0.001** | **-0.07**  **P<0.001** | **-0.24**  **P<0.001** |
| **All**  ****** | **0.50**  **P<0.001** | **0.62**  **P<0.001** | **0.52**  **P<0.001** | **0.51**  **P<0.001** | **-0.32**  **P<0.001** | **-0.43**  **P<0.001** | **-0.46**  **P<0.001** | **-0.36**  **P<0.001** | **-0.21**  **P<0.001** | **-0.21**  **P<0.001** | **-0.11**  **P<0.001** | **0.10**  **P<0.001** | **0.26**  **P<0.001** | **-0.29**  **P<0.001** | **0.02**  **P=0.23** | **0.23**  **P<0.001** | **-0.30**  **P<0.001** |

Supplementary Table S8: Voxelwise correlation analysis between tumour diffusion metrics (MD, FA) and DCE-MRI derived microvascular parameters (v_e_, K^trans^ and v_p_)

Voxelwise correlation coefficient derived using either all patient datasets (*) or three patient datasets (**) with complete follow-up imaging (patient 3, 4 and 5). Spearman’s rho shown.

|  | **MD: v_e_** | | | **MD: K^trans^** | | | **MD: v_p_** | | | **FA: v_e_** | | | **FA: K^trans^** | | | **FA: v_p_** | | |
| --- | --- | --- | --- | --- | --- | --- | --- | --- | --- | --- | --- | --- | --- | --- | --- | --- | --- | --- |
|  | **Pre** | **2 weeks** | **6 mon** | **Pre** | **2 weeks** | **6 mon** | **Pre** | **2 weeks** | **6 mon** | **Pre** | **2**  **weeks** | **6**  **mon** | **Pre** | **2**  **weeks** | **6**  **mon** | **Pre** | **2**  **weeks** | **6**  **mon** |
| 1 | -0.39  P<0.001 | -0.53  P<0.001 |  | -0.39  P<0.001 | -0.30  P<0.001 |  | -0.47  P<0.001 | -0.15  P=0.002 |  | 0.22  P<0.001 | 0.18  P<0.001 |  | 0.26  P<0.001 | 0.17  P=0.003 |  | 0.33  P<0.001 | 0.20  P<0.001 |  |
| 2 |  | -0.43  P<0.001 | 0.04  P=0.29 |  | -0.18  P<0.001 | -0.23  P<0.001 |  | 0.09  P=0.01 | 0.05  P=0.22 |  | 0.31  P<0.001 | 0.05  P=0.19 |  | 0.11  P=0.004 | 0.42  P<0.001 |  | -0.17  P<0.001 | 0.18  P<0.001 |
| 3 | -0.43  P<0.001 | -0.43  P<0.001 | -0.44  P<0.001 | 0.06  P=0.19 | -0.22  P<0.001 | -0.57  P<0.001 | 0.08  P=0.04 | 0.008  P=0.85 | -0.48  P<0.001 | 0.58  P<0.001 | 0.45  P<0.001 | 0.39  P<0.001 | -0.19  P<0.001 | 0.20  P<0.001 | 0.52  P<0.001 | -0.27  P<0.001 | -0.03  P=0.51 | 0.54  P<0.001 |
| 4 | -0.26  P<0.001 | -0.34  P<0.001 | -0.13  P<0.001 | 0.02  P=0.49 | 0.12  P<0.001 | -0.46  P<0.001 | -0.14  P<0.001 | -0.06  P=0.07 | -0.31  P<0.001 | 0.13  P<0.001 | 0.08  P<0.001 | -0.003  P=0.92 | -0.11  P<0.001 | -0.26  P<0.001 | 0.42  P<0.001 | -0.14  P<0.001 | -0.20  P<0.001 | 0.43  P<0.001 |
| 5 | -0.45  P<0.001 | -0.36  P<0.001 | -0.26  P<0.001 | -0.30  P<0.001 | -0.40  P<0.001 | -0.04  P=0.25 | -0.09  P=0.009 | -0.25  P<0.001 | -0.07  P=0.03 | 0.26  P<0.001 | 0.21  P<0.001 | -0.03  P=0.44 | 0.10  P=0.003 | 0.19  P<0.001 | -0.07  P=0.03 | 0.14  P<0.001 | 0.22  P<0.001 | 0.15  P<0.001 |
| **All**  ***** | **-0.38**  **P<0.001** | **-0.27**  **P<0.001** | **-0.15**  **P<0.001** | **0.05**  **P=0.01** | **-0.03**  **P=0.11** | **-0.30**  **P<0.001** | **-0.05**  **P=0.008** | **0.04**  **P=0.009** | **-0.21**  **P<0.001** | **0.30**  **P<0.001** | **0.02**  **P=0.17** | **0.06**  **P<0.001** | **-0.07**  **P<0.001** | **-0.06**  **P<0.001** | **0.26**  **P<0.001** | **-0.02**  **P=0.30** | **0.08**  **P<0.001** | **0.26**  **P<0.001** |
| **All**  ****** | **-0.50**  **P<0.001** | **-0.44**  **P<0.001** | **-0.20**  **P<0.001** | **0.12**  **P<0.001** | **0.005**  **P=0.81** | **-0.31**  **P<0.001** | **0.09**  **P<0.001** | **0.21**  **P<0.001** | **-0.26**  **P<0.001** | **0.39**  **P<0.001** | **0.27**  **P<0.001** | **0.04**  **P=0.04** | **-0.12**  **P<0.001** | **0.03**  **P=0.16** | **0.19**  **P<0.001** | **-0.12**  **P<0.001** | **-0.006**  **P=0.79** | **0.26**  **P<0.001** |

**Supplementary Table S9: Temporal changes in extra- and intracellular sodium population**

| **Patient** | **Pre / 2 weeks** | | | | | **Pre / 8 weeks** | | | **Pre / 6 months** | | | | |
| --- | --- | --- | --- | --- | --- | --- | --- | --- | --- | --- | --- | --- | --- |
|  | **↑**  **TSC** | **Extra-** | | **Intra-** | | **↑**  **TSC** | **Extra-** | **Intra-** | **↑**  **TSC** | **Extra-** | | **Intra-** | |
|  |  | **↑TSC:**  **↑MD** | **↑TSC:**  **↑ve** | **↑TSC:**  **↓MD** | **↑TSC:**  **↓ve** |  | **↑TSC:**  **↑MD** | **↑TSC:**  **↓MD** |  | **↑TSC:**  **↑MD** | **↑TSC:**  **↑ve** | **↑TSC:**  **↓MD** | **↑TSC:**  **↓ve** |
| 1 | **72.9** | 53.8 | 68.7 | 19.0 | 4.16 | **69.4** | 44.2 | 25.2 |  | | | | |
| 3 | **98.7** | 31.2 | 51.2 | 67.5 | 47.4 |  |  | | **99.4** | 74.6 | 6.52 | 25.0 | 92.9 |
| 4 | **100** | 60.5 | 66.6 | 39.5 | 33.4 | **98.2** | 49.5 | 48.7 | **99.7** | 87.8 | 37.8 | 11.9 | 61.9 |
| 5 | **52.1** | 26.9 | 14.6 | 25.2 | 37.4 | **16.9** | 10.7 | 6.18 | **96.4** | 88.3 | 17.2 | 8.13 | 79.2 |
| **All*** | **80.5** | 43.8 | 47.8 | 36.7 | 32.7 | **62.0** | 33.9 | 28.1 | **98.5** | 85.1 | 23.7 | 13.4 | 74.8 |
| **Mean**  **(S.D)**  ****** | **80.9**  **(22.9)** | **43.1**  **(16.5)** | **50.3**  **(25.0)** | **37.8**  **(21.6)** | **30.6**  **(18.6)** | **61.5**  **41.2)** | **34.8**  **(21.0)** | **26.7**  **(21.3)** | **98.5**  **(1.82)** | **83.5**  **(7.77)** | **20.5**  **(15.9)** | **15.0**  **(8.85)** | **78**  **(15.5)** |
| **P value** | **Extra- vs intra-**  **(TSC: MD)** | | | **P<0.001** | | **P<0.001** | | | **P<0.001** | | | | |
|  | **Extra-vs intra-**  **(TSC: v_e_)** | | | **P<0.001** | |  | | | **P<0.001** | | | | |

Percentage of voxels at each post-treatment timepoint displaying increased TSC, mean diffusivity (MD) and v_e_ relative to pre-treatment values shown. Percentage of all voxels demonstrating increased TSC, MD and v_e_ shown (*) alongside mean voxel % (± SD) across the 4 tumours studied (**).

P value shown is for comparison of all tumour voxels displayed an increase in either the extracellular or intracellular sodium population (one-sample test of proportions).

**Supplementary Table S10: Temporal changes in DCE-MRI microvascular parameters in tumour voxels displaying ↑TSC and ↑MD**

Percentage of either all tumour voxels or tumour voxels with increased TSC or MD at each post-treatment timepoint displaying increased increased v_e_, K^trans^ or v_p_ relative to pre-treatment values. Percentage of all tumour voxels demonstrating increased TSC/MD and v_e_, K^trans^ or v_p_ shown (*) alongside mean voxel % (± SD) across the 4 tumours studied (**).

| **Patient** | **Pre/2 weeks** | | | | | | | | | **Pre / 6months** | | | | | | | | |
| --- | --- | --- | --- | --- | --- | --- | --- | --- | --- | --- | --- | --- | --- | --- | --- | --- | --- | --- |
|  | **All**  **tumour voxels** | | | **Tumour voxels**  **displaying ↑TSC** | | | **Tumour voxels**  **displaying ↑MD** | | | **All**  **tumour voxels** | | | **Tumour voxels displaying ↑TSC** | | | **Tumour voxels**  **displaying ↑MD** | | |
|  | **↑v_e_** | **↑K^trans^** | **↑v_p_** | **↑v_e_** | **↑K^trans^** | **↑v_p_** | **↑v_e_** | **↑K^trans^** | **↑v_p_** | **↑v_e_** | **↑K^trans^** | **↑v_p_** | **↑v_e_** | **↑K^trans^** | **↑v_p_** | **↑v_e_** | **↑K^trans^** | **↑v_p_** |
| **1** | 95.6% | 99.3% | 57.9% | 94.1% | 99.7% | 52.0% | 97.5% | 99.1% | 61.1% |  | | | | | | | | |
| **3** | 52.4% | 61.3% | 88.2% | 52.0% | 61.4% | 88.2% | 50.3% | 61.1% | 88.0% | 7.35% | 1.16% | 12.8% | 6.99% | 1.17% | 12.8% | 5.25% | 1.57% | 9.19% |
| **4** | 66.6% | 20.2% | 66.2% | 66.6% | 20.2% | 66.2% | 59.6% | 15.9% | 63.9% | 37.8% | 6.11% | 3.01% | 37.9% | 5.84% | 2.82% | 38.6% | 4.97% | 2.10% |
| **5** | 27.9% | 61.0% | 35.6% | 28.1% | 60.0% | 34.8% | 32.9% | 62.6% | 41.3% | 18.6% | 35.8% | 33.8% | 17.9% | 35.7% | 33.1% | 17.7% | 37.1% | 31.3% |
| **All*** | **56.3%** | **52.3%** | **59.2%** | **59.3%** | **48.3%** | **62.6%** | **58.9%** | **51.1%** | **59.6%** | **24.3%** | **16.2%** | **16.6%** | **24.1%** | **15.8%** | **16.0%** | **24.4%** | **16.9%** | **14.8%** |
| **Mean**  **(S.D)**  ****** | **60.6%**  **(28.3)** | **60.5%**  **(32.3)** | **62.0%**  **(21.7)** | **60.2%**  **(27.6)** | **60.3%**  **(32.5)** | **60.3%**  **(22.6)** | **60.1%**  **(27.3)** | **59.7%**  **(34.1)** | **63.6%**  **(19.1)** | **21.3%**  **(15.4)** | **14.4%**  **(18.7)** | **16.5%**  **(15.7)** | **20.9%**  **(15.7)** | **14.2%**  **(18.7)** | **16.2%**  **(15.4)** | **20.5%**  **(16.9)** | **14.5%**  **(19.6)** | **14.2%**  **(15.2)** |
